# Wireless inertial measurement of head kinematics in freely-moving rats

**DOI:** 10.1038/srep35689

**Published:** 2016-10-21

**Authors:** Matthieu O. Pasquet, Matthieu Tihy, Aurélie Gourgeon, Marco N. Pompili, Bill P. Godsil, Clément Léna, Guillaume P. Dugué

**Affiliations:** 1 Institut de Neurobiologie de la Méditerranée (INMED), Institut National de la Santé et de la Recherche Médicale (Inserm), U901, Marseille, France; 2 École Normale Supérieure, PSL Research University, Centre National de la Recherche Scientifique (CNRS) UMR8197, Inserm, U1024, Paris, France; 3 Centre de Psychiatrie et Neurosciences (CPN), Inserm, U894, Paris, France; 4 Collège de France, Centre Interdisciplinaire de Recherche en Biologie (CIRB), CNRS UMR7241, Inserm, U1050, Paris, France

## Abstract

While miniature inertial sensors offer a promising means for precisely detecting, quantifying and classifying animal behaviors, versatile inertial sensing devices adapted for small, freely-moving laboratory animals are still lacking. We developed a standalone and cost-effective platform for performing high-rate wireless inertial measurements of head movements in rats. Our system is designed to enable real-time bidirectional communication between the headborne inertial sensing device and third party systems, which can be used for precise data timestamping and low-latency motion-triggered applications. We illustrate the usefulness of our system in diverse experimental situations. We show that our system can be used for precisely quantifying motor responses evoked by external stimuli, for characterizing head kinematics during normal behavior and for monitoring head posture under normal and pathological conditions obtained using unilateral vestibular lesions. We also introduce and validate a novel method for automatically quantifying behavioral freezing during Pavlovian fear conditioning experiments, which offers superior performance in terms of precision, temporal resolution and efficiency. Thus, this system precisely acquires movement information in freely-moving animals, and can enable objective and quantitative behavioral scoring methods in a wide variety of experimental situations.

Inertial sensing microelectromechanical systems have opened new avenues for the measurement of body kinematics. Their small form factor has allowed the design of wearable inertial measurement units (IMUs) that can track linear accelerations and angular rates in three dimensions from specific body segments. Clinical applications of IMUs include the diagnosis of balance and gait disorders^1^,^2^,^3^ and the analysis of motor symptoms in degenerative diseases such as Parkinson’s disease^4^. Ambulatory systems containing IMUs have been designed for detecting falls in the elderly^5^,^6^ and for guiding navigation in the sight-impaired^7^. IMUs also offer an efficient methodology for monitoring animal behavior. In animal husbandry, IMUs provide quantitative tools for general health assessment, for monitoring feeding behaviors and for estimating daily walking distances^8^,^9^,^10^. In the wild, IMUs have been used to study the behavior of more than a hundred species of marine, terrestrial and flying animals^11^,^12^,^13^,^14^,^15^,^16^,^17^,^18^. In the laboratory, IMUs offer a way to obtain accurate measurements of motor responses and locomotor behavior in various animal models^19^,^20^,^21^,^22^,^23^. They could also advantageously replace tedious and subjective observational scoring techniques in a wide range of experimental situations, as shown for the automatic detection of arousal states and behavioral sequences such as grooming, eating and rearing^24^,^25^.

Measuring precisely-timed motor responses is highly relevant to many research domains in the neurosciences, particularly when combined with electrophysiological recordings. In some cases, such as vestibular research, IMUs have the potential to enable new approaches. Previous vestibular research has focused mainly on understanding how the brain processes passively-experienced head movements, yet recent studies suggest that self-generated head movements are processed in a different way^26^, at frequency ranges well above those examined so far^27^,^28^. In future studies, IMUs will be essential for assessing the kinematics of natural, voluntary head movements, for studying how the corresponding vestibular signals are processed centrally, and ultimately for applying this knowledge to the design of vestibular prostheses^29^,^30^,^31^.

The possibilities of inertial measurements on small laboratory animals, such as rats and mice, depend critically upon the availability of the appropriate IMUs. For many applications, lightweight wireless solutions are preferable over tethered measurement systems, as cables may hinder animal movements. In addition, laboratory applications may require real-time interactions with the IMU to implement low-latency data timestamping or motion-triggered experiment control. Here we describe a small wireless IMU that can be assembled using off-the-shelf components and be attached to a rat’s head with a standard surgical procedure. Our IMU can broadcast inertial signals sampled at 300 Hz over a range of 10 m, and features advanced bidirectional synchronization capabilities. In this paper, we describe the architecture and main features of the device and provide examples illustrating its usefulness for tracking head movements and posture in freely moving rats. We propose in particular a novel method for quantifying behavioral freezing during Pavlovian fear conditioning experiments, that outperforms current scoring techniques. These examples demonstrate that our system has the potential to considerably simplify and refine the acquisition of movement information in a variety of experimental paradigms.

## Results

### Design of the wireless inertial sensing device

**Figure 1:**
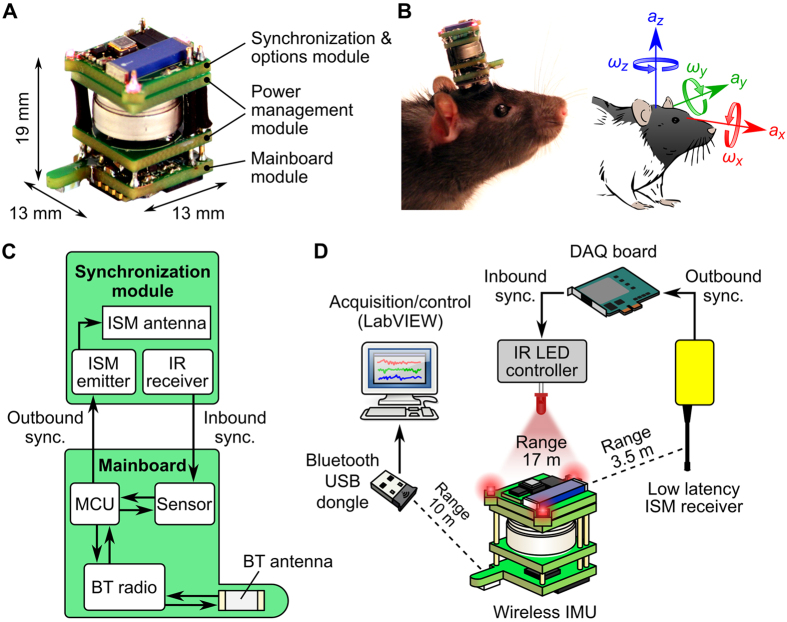
Overview of the wireless inertial measurement system. (**A**) Inertial measurement unit (IMU) and its main components. (**B**) Left: Photograph of an adult rat wearing the IMU. Right: sketch showing the directions of linear accelerations (*a*_*x*_, *a*_*y*_, *a*_*z*_) and angular velocities (*ω*_*x*_, *ω*_*y*_, *ω*_*z*_) measured by the sensor, with respect to the animal’s head. (**C**) Simplified diagram of the circuit managing the acquisition of inertial data and inbound/outbound synchronization on the IMU. (**D**) Schematic of the whole system, that comprises an IMU, a PC equiped with a Bluetooth USB dongle, a data acquisition (DAQ) board, a custom IR LED controller for inbound synchronization and a custom low-latency ISM receiver for outbound synchronization. The transmission range is indicated for each wireless communication channel. BT: Bluetooth; IR: infrared; ISM: industrial, scientific, medical; MCU: microcontroller unit.

Our system is composed of a battery-powered IMU (Fig. 1A), two external synchronization modules (Fig. 1D), a battery charger (Fig. S1A) and a client software. A lightweight (6 g with battery), compact (13 × 13 × 19 mm) and cost-effective IMU design was achieved by selecting off-the-shelf electronic components available in small packages. The IMU can easily fit onto a rat’s head (Fig. 1B) and could be further miniaturized to fit onto a mouse (see Discussion). Communication with the IMU is implemented via Bluetooth, a reliable and widespread wireless communication technology that offers an excellent trade-off in terms of performances and integrability (see Discussion). Our IMU can thus be controlled over a distance of 10 m from any PC with integrated Bluetooth support or equipped with a standard Bluetooth USB dongle.

#### Transmission delays

**Figure 2:**
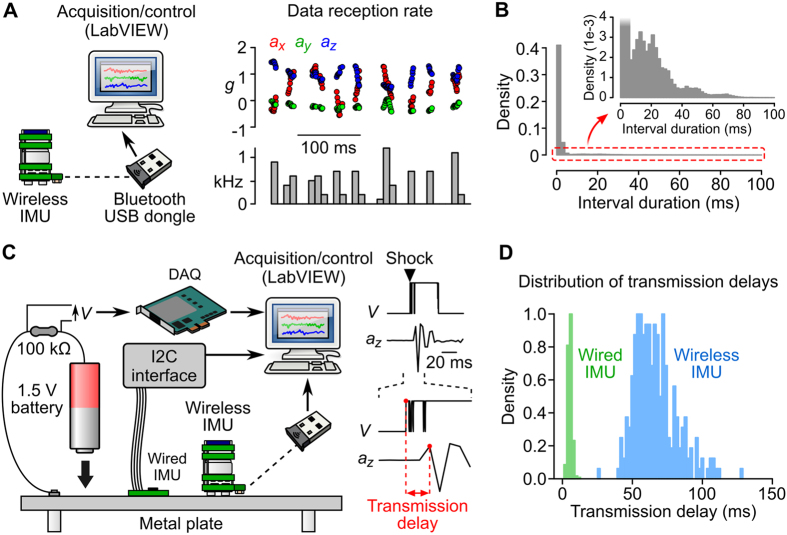
Bluetooth transmission of inertial data. (**A**) Left: schematic of the recording configuration. Right, top: accelerometer data along the 3 axes (*a*_*x*_, *a*_*y*_ and *a*_*z*_) were plotted against their reception time by the client software. Note the presence of occasional large gaps between successive data frames. Right, bottom: corresponding instantaneous data reception rate (bin = 10 ms). (**B**) Density histogram of time intervals between successive data frames in a 1 h recording. Inset: magnified view of the area delimited by a red dotted line. Note the presence of long intervals (>10 ms). (**C**) Left: schematic of the experiment used to measure transmission delays. A 1.5 V battery was hit against a metal plate, creating a voltage difference across a resistor (recorded using a DAQ) and a mechanical vibration (recorded using the wireless IMU or a wired IMU). Right: For each shock (here one shock is shown as an example), the first points significantly deviating from baseline were identified in the electrical (*V*, top) and inertial (*a*_*z*_, bottom) signals. The interval between the two points (red points in blown up traces) was taken as a measure of the data transmission delay. (**D**) Normalized histograms of transmission delays for the wired IMU (green) and the wireless IMU (blue), calculated using a total of 250 shocks (bin = 2 ms).

The acquisition and transmission of inertial data follow a framed traffic logic: at each acquisition cycle, information from the sensor is read, formatted into packets and sent to the Bluetooth radio. To guarantee data integrity, we selected a Bluetooth profile relying on ACL (Asynchronous Connection-Less), a protocol in which lost or corrupt data are retransmitted, introducing delays and jitters in data transmission. As a consequence, data frames are transmitted with a non-deterministic latency. When measuring data frame reception time, we observed that data were indeed received at an irregular rate (Fig. 2A), with 15% of inter-frame intervals exceeding 3 ms (calculated from a 1 h recording; Fig. 2B). To measure the delay between mechanical transduction by the sensor and data reception by the client software (transmission delay), we simultaneously generated a mechanical vibration and an electric signal by touching a 1.5 V battery to a metal plate (Fig. 2C; see Methods). Transmission delays, measured as the lag between the electrical and inertial signals for each contact, ranged from 26 to 128 ms (average delay: 66.6 ± 15.3 ms, n = 250 contacts; Fig. 2D). As a comparison, the average transmission delay measured with a wired IMU (see Methods) was 12.5 times shorter (average delay: 5.3 ± 1.5 ms, ranging 1.2–12.1 ms, n = 250 contacts; Fig. 2D). In conclusion, the use of a Bluetooth data transmission protocol does not allow timestamping of the acquisition of inertial data with a precision better than 50 ms, a situation that might be problematic for applications requiring precisely timed recordings. In addition, the measured transmission delays might not be adequate for implementing low-latency motion-triggered applications. These two issues are addressed in the following paragraph.

#### Low-latency synchronization functions

**Figure 3:**
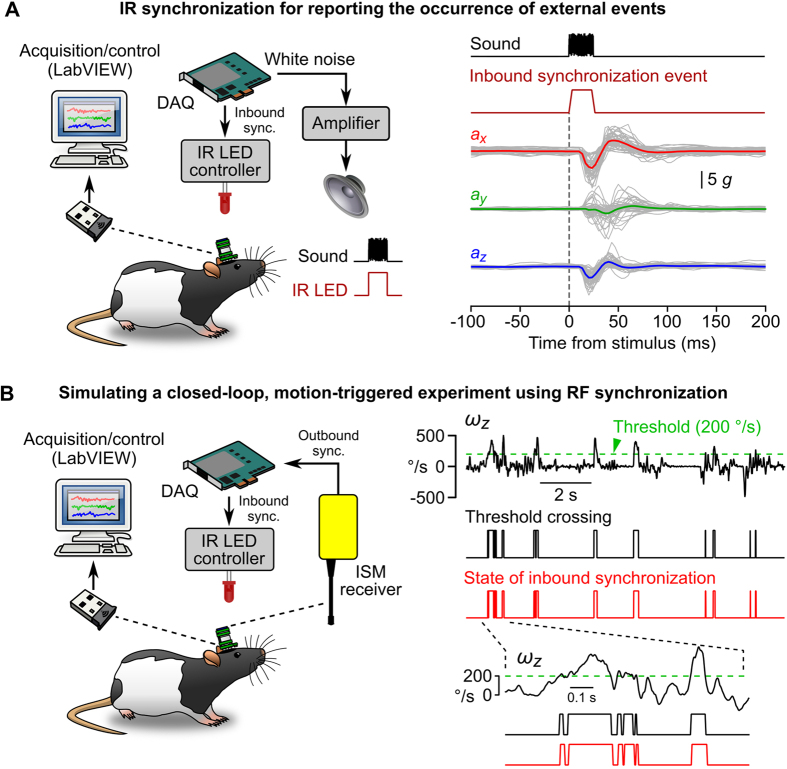
Bidirectional synchronization using two separate wireless channels. (**A**) Left: schematic of the acoustic startle experiment. Bursts of white noise (25 ms) were generated by a DAQ and played by a loudspeaker. At the onset of each stimulus, a synchronous 5 V signal was used to gate the emission of an IR synchronization signal. Right: startle responses recorded in one animal. Linear acceleration along the 3 axis (*a*_*x*_, *a*_*y*_ and *a*_*z*_) was aligned on the onset of the IR event and averaged. Superimposed gray lines represent individual sweeps and color lines represent average linear accelerations. (**B**) Left: schematic of the closed-loop, motion-triggered experiment. The IMU was configured to emit RF power when yaw angular speed towards the left exceeded 200°/s. The output of the RF receiver was monitored through the digital input channel of a DAQ and conditioned the execution of an analog output task. The analog output was a simple 5 V command that was used to gate the emission of IR signals. Right: Yaw angular velocity (*ω*_*z*_) and outbound/inbound synchronization information recorded by the IMU during a 12 s period. The dashed green line represents the angular velocity threshold above which RF power was emitted by the IMU. The black and red lines below represent the states of the booleans reporting the detection of an angular velocity value exceeding threshold (Threshold crossing, black) and the presence of an IR signal (State of inbound synchronization, red).

Low-latency inbound synchronization was implemented using an infrared (IR) communication channel (see Methods). The IMU’s circuit was designed such that the detection of IR signals is recorded directly by the sensor and transmitted together with inertial data (Fig. 1C). Therefore IR synchronization can be used to perform accurate data timestamping, a strategy useful for recording the occurrence of external events. We illustrated this by recording responses to startling acoustic stimuli in freely moving rats (see Methods). The acoustic startle reflex corresponds to involuntary muscle contractions in response to unexpected and loud auditory stimuli, typically occurring with a latency of 5–15 ms^32^. In our experiment, brief white noise auditory stimuli associated with IR signals were delivered randomly. IR synchronization events recorded by the IMU were used for offline realignement of inertial data on the onset of auditory stimuli. Startle responses were visible as sharp biphasic deflections of head linear acceleration in all 3 axes (Fig. 3A). The response magnitude was measured as the amplitude of the first peak relative to baseline (3.33 ± 1.37 *g* for *a*_*x*_ and 2.11 ± 0.86 *g* for *a*_*z*_, n = 4 rats) and its latency was measured as the time of the first point significantly deviating from baseline (11.7 ± 1.9 ms for both *a*_*x*_ and *a*_*z*_, n = 4 rats). This experiment shows that IR synchronization provides a way of accurately quantifying motor responses evoked by external stimuli. Data timestamping can also be employed for precisely interpolating the absolute acquisition time of individual data frames, simply by connecting the IR emitter to an external reference clock (see Fig. S4 and § 1.1 of the Supplementary Methods). If necessary, the occurrence of external events could then be recorded by a third-party acquisition system synchronized with the same external clock.

The microcontroller present in the IMU (see Methods) can be programmed to perform simple real-time calculations such as the detection of threshold crossing, a function that can be used to generate motion-triggered events (for closed-loop electrical or optogenetic stimulation, or for triggering specific events in a behavioral conditioning experiment). Triggers should ideally be generated from motion data with a low and constant latency. As shown above (Fig. 2D), a Bluetooth radio link is not suited for applications requiring low-latency, deterministic data transmission. We solved this issue by designing an independent radio frequency (RF) outbound synchronization channel (see Methods). Like for the inbound IR channel, the state of the RF channel is recorded in a boolean in each data frame. To simulate a closed-loop, motion-triggered experiment, we set the IMU to emit an RF signal when the yaw angular velocity *ω*_*z*_ exceeded a defined threshold, and configured a DAQ board to play an analog waveform every time an RF event was detected by the receiver (see Methods). In the present case, the waveform was a 5 V step used to gate the emission of an IR signal (Fig. 3B). The system was tested in a rat exploring a circular arena. All episodes during which *ω*_*z*_ exceeded the defined threshold were correctly detected by the microcontroller and written in the inertial data flow (n = 321 events detected in 450 s; average event duration = 44.1 ± 45.1 ms) and almost all of them (96.3%) appeared as IR synchronization events in the IMU data frames (Fig. 3B). In 96.4% of the cases, the IR synchronization event was recorded by the IMU exactly one frame after threshold crossing, indicating that the feedback loop was completed in less than 3.3 ms (the duration of an acquisition cycle).

### Inertial measurements in freely moving rats

#### Head kinematics during natural movements

**Figure 4:**
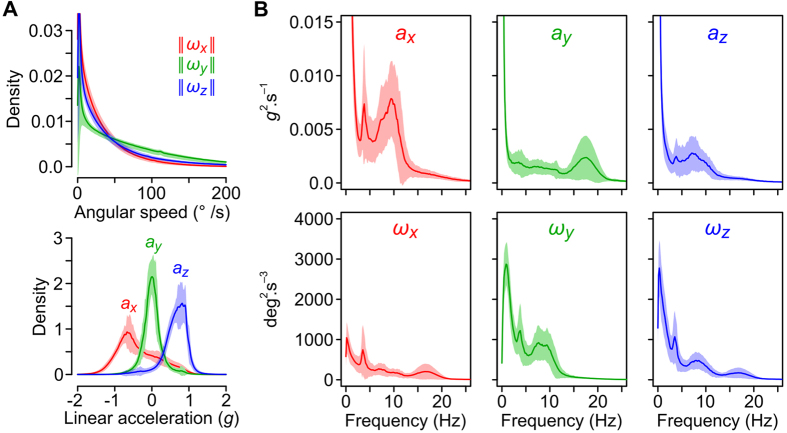
Head kinematics during free ambulation in rats. (**A)** Top: average density histogram of head angular speeds (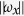, 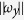, 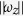). Bottom: average density histogram of the head linear accelerations. The different mean values of *a*_*x*_, *a*_*y*_ and *a*_*z*_ reflect the influence of gravity. (**B**) Average power spectral density histograms for accelerometer and gyroscope data. Average histograms in (**A,B**) were calculated from 19 rats.

Our IMU offers a way to measure the kinematics of head movements during normal activity in rats. We observed rapid head rotations (>50°/s) to be more frequent in the pitch axis (*ω*_*y*_) than in the roll and yaw axes (*ω*_*x*_ and *ω*_*z*_, respectively; Fig. 4A). The spectral power of natural head movements was concentrated at frequencies <20 Hz, with noticeable peaks at around 4, 8 and 15–20 Hz (Fig. 4B). During active exploration, head angular velocities reached values up to 500°/s (in particular during vigorous orientation movements), and displayed periods of 10 Hz oscillations, potentially reflecting rhythmic oro-facial activity such as sniffing (Movies S1 and S2). Large oscillations around 4 Hz were also evident during body grooming (Movie S3). Overall, accelerometer and gyroscope data provide a wealth of information that contains clear signatures of the different types of ongoing motor activities. In the future, this type of data should be appropriate to fuel robust behavioral scoring algorithms, as shown by pioneering studies that have employed wired accelerometers in mice and rats^24^,^25^.

#### Measuring head posture in freely moving rats

**Figure 5:**
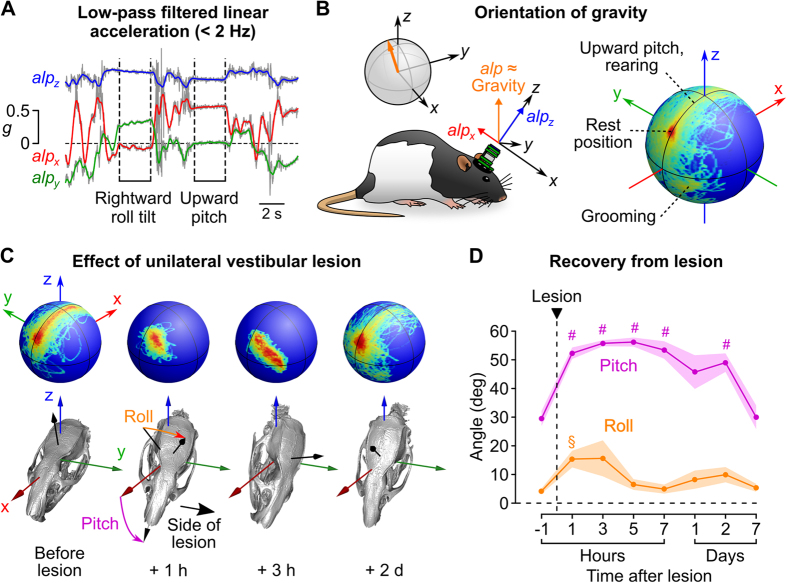
Tracking head posture in freely moving rats after a unilateral vestibular lesion. (**A**) Example traces showing the slow (<2 Hz) components of linear acceleration (*alp*_*x*_, *alp*_*y*_ and *alp*_*z*_, colored lines) superimposed on the raw linear acceleration (gray lines). These filtered signals capture the variations of linear acceleration due to reorientations of the head relative to gravity. (**B**) Left: *alp* (the vector defined by *alp*_*x*_, *alp*_*y*_ and *alp*_*z*_) is an approximation of gravity in the sensor’s coordinates. Right: density map of the different orientations of *alp* encountered during a 45 min recording session. (**C**) Example showing the effect of a unilateral vestibular lesion in one rat. Top: *alp* orientation density maps. Bottom: corresponding average head postures. (**D**) changes in the average pitch and roll angles (as defined in **C**) across sessions. Shaded areas represent the SEM. Symbols indicate statistically significant differences from angle values calculated before lesion (^§^*p* < 0.01, ^#^*p* < 0.001, paired t-test).

Because accelerometers are sensitive to gravity, our IMU can be used to monitor head posture. Head orientation relative to gravity (head tilt) can change, for example, as a result of a peripheral vestibular lesion. In various animal models including rats, head tilt is a key parameter for assessing the severity of postural deficits induced by unilateral vestibular lesion (UVL) and for studying the plasticity mechanisms that underlie the recovery of normal posture^33^,^34^. Changes in head tilt following UVL have traditionally been quantified with photographs of lightly restrained animals or direct observations logged on a discrete scale^34^,^35^, a procedure that can yield imprecise measurements and may not detect small tilt angles. Our IMU can facilitate this type of experiment by enabling accurate measurements of head tilt in unrestrained animals. Because natural head movements in rats are typically brief, the low frequency component of linear acceleration should provide a reliable approximation of gravitational acceleration in the sensor’s coordinates. To confirm this, we ran our intertial data through an orientation filter algorithm^36^ in order to estimate the gravitational component of linear acceleration (see § 1.4.1 of the Supplementary Methods). As shown in Fig. S5, gravitational acceleration accounted for most of the raw linear acceleration at frequencies below 1–2 Hz. We therefore used the low-pass filtered linear acceleration vector (*a*_*lp*_, calculated with a cutoff frequency of 2 Hz) as a proxy measure of gravity (Fig. 5A). A “gravity orientation density map” can be obtained by color coding the different orientations of *a*_*lp*_ on a sphere depending on how frequently they were encountered (see § 1.4.2 of the Supplementary Methods). This type of plot provides a snapshot of the different head postures of a rat during natural behavior, with different domains of the sphere corresponding to different types of motor activities (Fig. 5B). Rearing and grooming, for example, are associated with specific head orientations (Movie S3 and Movie S4). The calculated maps were very similar between rats (Fig. S6), with a mean correlation coefficient of 0.78 ± 0.14 (n = 19 rats).

To assess the system’s ability to discriminate changes in head tilt in freely moving animals, we performed UVLs in eight rats (see Methods). This manipulation narrowed the distribution of *a*_*lp*_ orientations, indicating that head mobility was decreased, and shifted it in a direction opposite to the lesion side, corresponding to a roll tilt towards the lesion side (Figs 5C and S7). The effect of the lesion could be quantified for each rat by analyzing the changes in the components of *a*_*lp*_ (Fig. S8). In addition, the average *a*_*lp*_ vector could be used to calculate the average head orientation and visualize it in 3D (Fig. 5C; see Supplementary Methods). Rats recovered a normal distribution of head postures within a week. Recovery curves were obtained by calculating the average roll and pitch angles of the head in successive recording sessions (Fig. 5D; see § 1.4.2 of the Supplementary Methods). These data show that inertial recordings offer a way of performing quantitative measures of head postures, that can be used to precisely monitor the effect of unilateral vestibular lesions.

#### Automatic scoring of behavioral freezing

For nearly 50 years researchers have used various forms of body immobility as a measure for estimating fear in rodents^37^. Initially, observational methods were the most commonly applied technique for assessing rat immobility^38^,^39^,^40^, yet various automated methods have also been developed, based on IR beam breaks^41^, changes in pixels in video recordings^42^ and load cell platform measurements^43^. Miniaturized IMUs offer a powerful alternative to these approaches that could potentially outperform previous methods in terms of precision, temporal resolution and efficiency.

**Figure 6:**
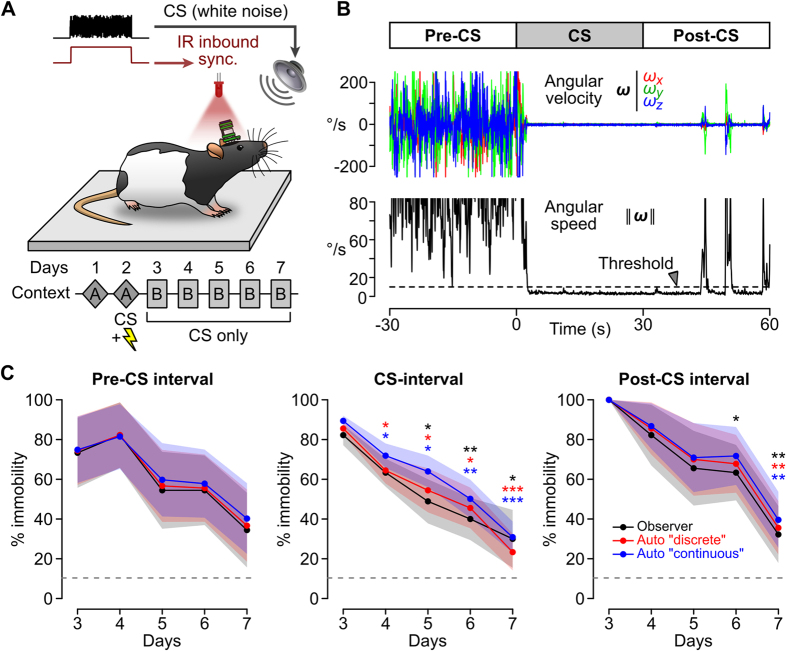
Automatic detection of behavioral freezing in a classical fear conditioning experiment. (**A**) Schematic of the experiment (see also Fig. S9C). The conditioned stimulus (CS) was a 30 s white noise. Fear conditioning (day 2, CS+ electric shock) and testing (days 3–7, CS only) occurred in two different contexts (**A**,**B**). The CS was presented 6 times during each of the 5 testing sessions, together with a synchronous IR signal that was recorded by the IMU (IR inbound sync.). (**B**) Example traces showing head angular velocities (top, colored traces) and angular speed (bottom, black trace) during a *trial*, defined as a period encompassing one CS presentation and the 30 s before (Pre-CS) and after (Post-CS). The dashed line represents an immobility detection threshold set at 12°/s. (**C**) Fear extinction profile for one rat. The average *per session* immobility scores (±SD as shaded area) were calculated using the observer’s data (black line) and the “discrete” automatic scoring method (red line) for the intervals before (Pre-CS), during (CS) and after (Post-CS) CS presentation. The fraction of time spent immobile was calculated using the “continuous” automatic scoring method (blue line). The horizontal dashed line represents the fraction of time spent immobile during the first exposure to context A (day 1). No significant differences were found between curves for a given extinction session (*p* always greater than 0.14, unpaired t-test). For days 4 to 7 (second to fifth extinction session), asterisks (color corresponding to the type of scoring technique) indicate whether the freezing score was significantly different from day 3 (first extinction session), with *p < 0.05, **p < 0.01 and ***p < 0.001 (unpaired t-test).

We evaluated the usefulness of the IMU for scoring behavioral freezing of rats in a Pavlovian fear conditioning experiment. We first characterized the noise in our system by recording data from an IMU placed on a mechanically isolated platform. In these measurements, the SD of the magnitude of the linear acceleration (respectively angular velocity) vector was 0.0089 *g* (respectively 0.103°/s), which represented respectively 3.78% and 0.10% of their SD measured during free ambulation (0.235 *g* and 106.8°/s respectively, n = 19 rats). These data show that angular velocity measurements are more appropriate than linear acceleration measurements for detecting motion. In our automated analysis, episodes of immobility were defined as periods during which angular speed (the magnitude of the angular velocity vector) was below a selected threshold (Fig. 6B).

Inertial data and video recordings were obtained for 4 rats during 5 daily fear extinction sessions following a fear conditioning session (see Methods and Fig. 6A). Each extinction session contained 6 CS presentations (30 s white noise cue). Behavioral freezing was scored from videos by an experienced observer implementing an instantaneous time sampling procedure^44^. This procedure consisted of the assessment of the state of the animal (1 = “freezing”, 0 = “no-freezing”) every 2 s, during *trials* which included the 30 s before (pre-CS), during (CS) and 30 s after (post-CS) each white noise presentation (Fig. S9C). With IMU data, for comparison, we implemented a “discrete” automatic scoring method that mimicked this procedure by comparing the local average angular speed to a threshold every 2 s (see Methods and Fig. S9A). Average *per trial* immobility scores were calculated using both methods, and compared by computing their correlation coefficient. Changing the duration of the observation window (from 0.1 to 2 s) did not strongly alter the correlation. In constrast, the correlation was highly dependent on the value of the detection threshold, and a maximal correlation of 0.98 was achieved for a threshold of 13 °/s (Fig. S9E).

Individual scores assigned by the observer and the algorithm for every observation window matched in 90.3% of the cases (n = 4878/5400 observations, calculated with the following algorithm parameters: window width = 0.5, threshold = 13°/s). “Error” cases (where a mismatch was found) were often associated to observation windows in which the average angular speed was close to the threshold (Fig. S10A), and were more frequently encountered during CS (14.2% of observations during CS vs 6.5% and 8.3% for pre-CS and post-CS intervals). When examining angular speed traces for error cases, it appeared that a large fraction of them corresponded to ambiguous situations, when the observer may have made his judgment shortly before or after a head movement (Fig. S10B).

An alternative observational scoring method consists of estimating the ratio of time spent freezing by measuring the onset and offset of freezing episodes using a stopwatch^45^. We implemented a similar “continuous” scoring method by calculating the fraction of the time during which angular speed fell below a threshold (Fig. S9B,D). This method was applied to the same 90 s intervals scored by the observer for each CS presentation, and the fraction of time spent below threshold was compared to the average immobility scores previously obtained by the observer using the instantaneous time sampling method. We found that a maximal correlation coefficient of 0.99 was achieved with a detection threshold of 12°/s (Fig. S9F).

Temporal fear extinction profiles for every rat were obtained by plotting the average immobility score (or the average fraction of time spent below threshold) in the pre-CS, CS or post-CS intervals for the 5 consecutive extinction sessions (see Fig. 6C for an example from one rat). No significant differences were found between extinction curves calculated using the three scoring methods (observer and automatic “discrete” or “continuous” methods, *p* always greater than 0.14 for all rats, unpaired t-test).

We conclude that automatic scoring methods based on a simple threshold crossing analysis of head angular speed yield results that are virtually indistinguisable from those obtained by an experienced observer. Because of their ease of implementation and rapidity, these methods have the potential to considerably facilitate and refine the scoring of behavioral freezing. The scoring of behavioral freezing by an observer is a time-consuming endeavor, and most typically freezing is only estimated during limited intervals of the testing session (such as during the CS presentation). Our device can precisely approximate human scoring, and it can be used to estimate behavioral freezing continuously throughout the testing session. Hence, more data can be collected with less labor on the part of the experimenter.

## Discussion

Because it allows quantitative and meaningful measurements of activity with a resolution that could not be achieved with direct observation, inertial motion sensing has become a widely recognized methodology for the study of animal behavior^11^,^12^,^13^,^14^,^15^,^16^,^17^,^18^. Untethered inertial measurements are typically performed at 1–100 Hz on relatively large animals using bulky telemetric devices or accelerometers coupled to onboard data-logging systems. Surprinsingly, inertial measurements on laboratory animals such as rats or mice remain relatively rare, and have mainly employed wired inertial sensors^19^,^20^,^22^,^23^. To our knowledge, there is currently no solution for real-time wireless monitoring of movements at high rate in freely-moving rats or mice. In this paper, we describe and benchmark a standalone platform for performing wireless inertial measurements of head movements in rats. The system was designed to satisfy the following constraints: lossless data transmission, sufficient acquisition rate, low power consumption, small hardware size and weight, low cost and ease of integration. For data transmission and communication with the IMU, Bluetooth appeared as an excellent trade-off. Bluetooth offers excellent hardware and software integrability, through the availability of miniature modules and of SPP (Serial Port Profile) for seamless serial link replacement. It allowed a rapid development using miniature off-the-shelf components while offering the necessary bandwidth and data integrity.

The one limitation of Bluetooth is the fact that it offers an asynchronous connection (Fig. 2D). This limitation was overcome by implementing real-time bidirectional communication through two separate channels: an IR channel for data timestamping, and an RF channel for motion-triggered applications (Fig. 3). The IR channel can be used to record the occurrence of external events (Fig. 3A) or to synchronize the acquisition of inertial data with an external clock (Fig. S4). Note that the RF channel could also be used to synchronize data acquisition with external devices. The IMU can for example be programmed to emit an RF signal every *N* acquisition cycles (*N* to be determined by the user; see for example § 1.2.2 in the Supplementary Methods). Owing to the sub-millisecond latency between the emission and detection of RF signals (Fig. S2B), the acquisition time of the corresponding data frames (identified by a change in the state of the RF channel) could be precisely measured. The acquisition time of other data frames could then be calculated using a simple linear interpolation. This strategy would allow the user to synchronize data acquisition with other devices while saving the IR channel for the detection of external events.

Overall, our system meets the requirements of most laboratory applications, and can easily be coupled with existing third-party acquisition and control devices. Therefore it makes wireless inertial recordings of head movements accessible to any laboratory working with freely moving animals with the size of a rat. Note that Bluetooth allows for multiple devices to operate simultaneously, allowing measurements on several animals at the same time.

We demonstrated the usefulness of our IMU in several experimental situations. In a first experiment, we showed how the IR synchronization channel can be used to align inertial data to the onset of a startling auditory stimulus and to calculate average startle responses (Fig. 3A). IR synchronization enables the characterization of any kind of stimulus-evoked head movement. It could thus be useful for quantifying motor responses evoked by external cues in a number of learning pardigms, or for characterizing motor responses evoked by electrical or optogenetic stimulations. IR data timestamping also enables the synchronization of IMU data sampling with other acquisition systems, and thus the analysis of the correlation between inertial data and other signals on a millisecond timescale.

We then simulated a closed-loop, motion-triggered experiment based on the onboard detection of threshold crossings (Fig. 3B). Coupling the delivery of stimuli to specific head movements expands the possibilities of operant conditioning experiments. Currently, these experiments rely on a relatively limited repertoire of actions, such as nose pokes or lever presses. The diversity of natural head movements, mostly along the pitch and yaw rotation axes, could provide alternate behavioral responses for such experiments.

In a separate experiment, we showed that our IMU can be used to perform quantitative measures of head posture in unrestrained animals and to monitor precisely the effect of a unilateral vestibular lesion (Fig. 5). Compared to traditional observational techniques that often employ subjective scoring on a discrete scale, the proposed method offers objective and quantitative measures on a continuous scale. It also provides a richer information on the state of the animal, by fully capturing the range of head postures encountered during free behavior. By enhancing the resolution of posturographic measurements in rats, this type of measurement might help to differentiate the effects of drugs designed to improve recovery after a vestibular trauma.

Finally, we showed that our IMU can facilitate considerably the quantification of behavioral freezing in a fear conditioning experiment (Fig. 6). The proposed method has a number of advantages compared to other automatic scoring methods, while offering the same level of concordance with a trained observer. Because movements are recorded directly from the animal’s head, our IMU provides more accurate information than any conventional actimetric device (based on IR beam breaking or video). Using a simple numerical simulation, we compared the performances of our IMU with the one of a high-resolution, high-rate motion capture system (see § 1.6 of the Supplementary Methods and Fig. S11 for detailed explanations). Assuming that the goal is to detect head rotations from a rat placed in a 1 m^2^ square area, our simulation shows that gyroscope signals acquired by the IMU are better at resolving small and rapid head rotations similar to the ones observed in freely moving rats (Fig. S11E–H). In terms of reliability and usability, our IMU also has a number of key advantages. A motion capture system could eventually be affected by cables connected to the animal, that might temporarily mask the reflectors or LEDs used for video tracking. Our IMU enables measurements in closed environment, with animals hidden behind walls (at the expense of IR synchronization using a single light source above the behavioral testing apparatus). Contrary to a video tracking system, the resolution of IMU measurements is not affected by a change in the size of the behavioral platform. Finally, the low cost of our system makes it a much cheaper solution than high-resolution motion tracking systems for quantifying behavioral activity (≃370 € versus several tens of thousands of euros).

Our wireless IMU provides an attractive solution for quantifying the movements of laboratory animals in various environments, from their homecage to complex mazes containing sheltered spaces. The quantification of natural head kinematics is particularly relevant to the field of vestibular research. A primary aim in this field is to understand how inertial motion signals transduced by vestibular organs are processed by the brain. A considerable literature has described how passively experienced head rotations and translations are encoded by central vestibular neurons, but the responses of these neurons to self-motion are still largely unknown^26^. The amplitude and frequency of the sinusoidal movements used in previous studies are typically low (±0.2 *g* for translations and ±30°/s for rotations, at 0.2–2 Hz) compared to the ranges experienced during self-motion^27^,^28^ (Fig. 4). Our system offers the possibility of characterizing the inertial signals detected by vestibular organs during self-motion, providing the foundation for exploring vestibular circuits in their normal operating conditions.

More generally, the data collected by our IMU contain a wealth of information on head movements and posture (Fig. 5 and Supplementary Movies). Previous studies have shown that 3-axis acceleration information is already useful for detecting and scoring various types of behavioral outputs^24^,^25^. Thus the 6-axis information provided by our IMU might help refine the automatic detection and quantification of behavioral outputs with a clear head movement signature, such as locomotion, sleep/wake cycles, rearing, grooming, eating or sniffing.

Our system could benefit from a number of achievable improvements. The size and weight of our IMU could be reduced. Especially, as we wished to use standard PCB manufacturing processes, we limited our design to 1.55 mm thick boards in 2 copper layers. The use of 0.2 mm thick, 4 layers boards would lead to a size reduction of at least 5.6 mm along the largest dimension and a weight reduction of 2.3 g. Extra weight could be saved by using a smaller and lighter battery such as the 201013HS10C (Full River Batteries, China). This would save an extra 1.2 g and 2.6 mm along the largest dimension, but would come at the price of a reduced battery life. Removing the synchronization/options board would save extra weight and would reduce the largest dimension by an additional 1.5 mm, while maintaining the core function of our device. By implementing these modifications and slightly redesigning our PCB to fit the new battery, our device could weight less than 2.5 g and measure 10 × 14 × 9 mm. Further miniaturization could be achieved by using a more compact Bluetooth module such as the PAN1315b (Panasonic, Japan) but would require important hardware and firmware redesign as well as possibly more expensive assembly techniques.

In addition to miniaturization, our system could evolve in a number of directions. One improvement would be to perform onboard calculation of absolute 3D orientation by implementing a sensor fusion algorithm. This would require either replacing our current microcontroller with a more powerful microprocessor, or using commercially available sensor units that already perform such calculations. Future versions of our IMU could also host more than one sensor. In our design, the microcontroller is programmed to make use of an I^2^C bus to communicate with the sensor. The I^2^C protocol allows one master (here the microcontroller) to communicate with several slaves (the sensors). Additional sensors could thus be easily wired to the existing I^2^C channel on the mainboard module and provide access to a wide range of variables such as biopotentials (electroencephalographic and electromyographic activity), barometric pressure and temperature (which can be used to monitor sniffing activity) or ultrasound waves (to detect vocalizations). Integrating additional sensors would require a reduction of the bandwidth currently allocated to inertial data, but a reduction of at least 50% (from 300 to 150 Hz) would be acceptable given the frequency range of natural head movements (Fig. 4B). Because our system uses a standard Bluetooth data link, client software could be implemented on various mobile devices such as tablets and smartphones. Inertial measurement could thus be performed rapidly and easily in various types of indoor and outdoor environments.

In conclusion, our system architecture lays the ground for implementing wireless inertial motion tracking at an affordable cost, and with advanced synchronization capabilities. It could also inspire the design of similar devices for performing wireless time-resolved measurements of other biologically-relevant parameters in freely moving laboratory animals.

## Methods

### Design and fabrication of the system

#### Inertial measurement unit

The IMU is composed of three modules on separate printed circuit boards (PCBs): a mainboard module, a power management module and a synchronization/options module (Fig. 1A).

The mainboard contains a digital 9-axis inertial sensor (MPU-9150, Invensense) that samples linear acceleration, angular velocity and magnetic field strength in three dimensions, a low-power programmable microcontroller (PIC16, Microchip) running a custom firmware and a Bluetooth radio, whose signal is transmitted through a tuned chip antenna. The microcontroller is programmed to handle the initialization and management of the sensor (via an I^2^C bus) and the radio, and to decode parameterization and operation commands received from the client software. Sensor data acquisition and Bluetooth data emission are scheduled by the microcontroller using interrupt mechanisms, in order to guarantee real-time performances of the system. The microcontroller firmware also implements simple data processing functions such as the detection of threshold crossing events in inertial data, which can be signaled via the emission of outbound RF signals.

The IMU is powered by a rechargeable Lithium-Ion battery (CP 1254, VARTA microbattery GMBH) inserted between the two PCBs composing the power management module (Fig. 1A). Electrical contacts are ensured by a metallized pad on one side, and a metallic spring on the other. The battery can be easily installed and removed manually. The power management module monitors the battery, switches the system on and off, and regulates the IMU supply voltage. It contains a voltage detector that switches the system off when the battery voltage reaches its lower limit, protecting it from excessive discharge. It also contains a magnetic latch that switches the system on and off by placing a small magnet near the IMU.

The synchronization/options module implements wireless low-latency, low-jitter bidirectional synchronization between the IMU and third-party devices. Inbound synchronization is used to timestamp IMU data frames using external triggers. Outbound synchronization is used to report the occurrence of movement-related events, such as the crossing of selected threshold values.

At the IMU level, inbound synchronization relies on the use of an integrated IR receiver that detects structured optical signals (light pulses in the 850–1080 nm range delivered at 38 kHz). Once activated, the receiver’s output is latched by a dedicated digital input channel of the sensor. The microcontroller interrogates the state of the sensor’s digital input register along with the inertial data registers at each acquisition cycle, thus inbound synchronization information is available in each data frame together and synchronized with inertial data. The latch mechanism guarantees that fast-occurring synchronization events (with a duration inferior to the microcontroller’s acquisition cycle duration) are not missed.

Outbound synchronization signals are computed by the microcontroller, fed to a low-power 433 MHz Industrial Scientific Medical (ISM) band emitter and radiated through its antenna (Fig. 1C). The ISM emitter is powered through the same supply line as the rest of the system, and thus benefits from the same power supply management functions (voltage regulation, on/off switching). While it also guarantees a compact design, this configuration powers the emitter with a supply voltage slightly inferior to that specified for the component (2.8 V instead of at least 3 V), which might explain the relatively limited transmission range that we measured (3.5 m). This range, however, is acceptable in the context of most laboratory applications. If necessary, solutions exist to detect RF signals over greater distances without increasing the device’s weight and volume, for example by combining several receivers.

The synchronization/options module also contains three low-power red LEDs (peak wavelength of 628 nm) for videotracking (Fig. 1A), which can be wirelessly switched on and off by the microcontroller upon request by the client software.

To achieve a compact device, the IMU was designed as a series of vertically stacked double-sided PCBs (produced by Eurocircuits, N.V., Belgium). Almost all components were surface-mount devices and were assembled in-house using a reflow soldering oven (FT02, CIF, France).

#### External synchronization modules

For inbound synchronization, we developed an IR emitter to activate the IR receiver located on the IMU’s synchronization/options module. The emitter contains a 38 kHz oscillator (based on a HCC4047BF, STMicroelectronics) gated by an external TTL signal and controlling a set of IR LEDs (TSAL6200, Vishay; peak wavelength = 940 nm) through a MOS transistor (IRF530, Vishay). The emission of IR signals is detected by the receiver with a latency of around 130 μs (Fig. S2A). The transmission range of the IR synchronization channel depends on the type and number of LEDs in the emitter, and on their drive current. For reference, the range obtained with one 940 nm LED powered with a drive current of 100 mA was 17 m.

The outbound synchronization receiver is a standalone module that detects RF signals sent by the IMU. It is a low-latency, low-jitter 433 MHz ISM band receiver designed for the transduction of RF signals into digital signals. Its architecture relies on an RF amplifier, a set of SAW filters, a logarithmic amplifier used as an RF power detector and a data slicing circuit performing signal digitization. The latency measured between the ISM emitter input on the IMU and the RF receiver output lies in the 33–48 μs range (Fig. S2B).

#### Battery charger

We developed an automatic Li-Ion battery charger that is fine-tuned to the specifications of our batteries. The charger contains five independent charging positions (Fig. S1A) so that batteries with different charge levels can be fully recharged at the same time. Each position contains an automatic Li-Ion battery charge management integrated circuit (Microchip Technology Inc.), that regulates the charge voltage and current, and manages the charge end so that the battery is optimally charged while remaining within its specifications. A thermal protection on each position ensures that the charge is stopped in case of battery over-heating. The charger can be powered using any computer USB port or USB power wall adapter.

The battery life was measured in different use-case examples. Each measurement was performed with a fully charged battery and consisted of streaming inertial data until the system was switched off by the voltage detector present on the power management module. The average recording durations in the different conditions tested are shown in Fig. S1B.

#### Software

The MCU firmware is written in C using the MPLAB X IDE suite and compiled using the XC8 C compiler (Microchip Technology Inc.). Real-time programming techniques such as interrupts have been used to guarantee the stability of the acquisition frequency. The firmware has been intensively tested to ensure data integrity and reliability.

The client software, written under LabVIEW (National Instruments), configures IMU parameters and acquires inertial and inbound/outbound synchronization data. The software allows the following operations: setting the sensitivity range of the accelerometers (from ±2 *g* to ±16 *g*) and gyroscopes (from ±250°/s to ±2000°/s), setting threshold values for the threshold crossing detection algorithm, starting and stopping data streaming, and controlling the LEDs.

### Inertial measurements of head movements in freely moving rats

#### Animals

All animals used in this study were adult male Long-Evans rats (250–300 g at surgery). Animals were housed individually in standard homecages maintained in standard laboratory conditions (12 h day/night cycle, ~21 °C with free access to food and water). The experimental procedures were conducted in conformity with the institutional guidelines and in compliance with French national and European laws and policies. All procedures were approved by the “Charles Darwin” Ethics Committee (project number 1334).

#### Magnetic head mount system

The IMU was secured on the animals’ heads by two pairs of disk neodymium magnets (S-06-03-N, Supermagnete.com). One pair was glued to the bottom face of the mainboard module, and the other one was cemented to the skull of the animal (see Supplementary Methods). Using this system, the IMU could be easily and rapidly snapped into position on the rat’s head before each experiment. This procedure usually switched the system on by activating the IMU’s magnetic latch. We found that the proximity of the magnets, once the IMU is in place, did not cause untimely on/off switching of the system. In addition, the system could still be turned on and off by placing a magnet near the IMU. The MPU-9150 inertial sensor also includes a 3-axis magnetometer. The magnetic field data were not used. However, the presence of the mounting magnets may prevent the normal functioning of the sensor’s magnetometer. A non-magnetic mounting system could be employed in order to preserve the integrity of magnetometer measurements if desired.

#### Acoustic startle experiment

Rats were equipped with the wireless IMU and placed inside a circular arena (118 cm diameter) with transparent plastic walls (30 cm high). The sensitivity of the IMU was configured remotely from the software interface (accelerometers: ±16 *g*; gyroscopes: ±1000°/s) and the acquisition of IMU data was initiated. A 25 ms analog waveform with uniform white noise distribution was generated under LabVIEW and played at random intervals (5–15 s) through the analog output of a DAQ board (PCIe-6353, National Instruments) connected to a loudspeaker via a custom amplifier (Fig. 3A, sound amplitude: 90–110 dBA). A square 5 V waveform with the same duration was played simultaneously via a second analog output channel, and fed to the gate input of the IR LED controller. A total of 30 acoustic stimuli were delivered per rat.

#### Motion-triggered, closed loop experiment using bidirectional synchronization

A rat carrying an IMU was placed in the same arena used for the acoustic startle experiment and was allowed to explore it for several minutes. The client software was used to set the sensor such that outbound RF power was emitted when yaw rotation speed towards the left exceeded 200°/s. RF signals were detected and digitized by the RF receiver, and fed to one of the digital inputs of a DAQ board. A custom LabVIEW program was configured to play an analog waveform every time a digital input high state was detected and halted it when the digital input channel returned to a low state. The analog output waveform was a simple 5 V step and was fed to the gate input of the IR LED contoller. Because the data transmitted by the IMU contains information on the states of the IR and RF synchronization channels, this configuration allowed us to estimate the duration of the feedback loop.

#### Unilateral vestibular lesion experiment

Unilateral excitotoxic lesions of the inner ear were obtained in eight rats using a procedure described earlier^35^. Rats were laid on their side under isoflurane anesthesia, placed under a surgical microscope, and 50 μL of a solution containing 50 nM of kainic acid (0222, Tocris) dissolved in physiological saline with 10 mg/mL benzyl alcohol (305197, Sigma; used to enhance round window permeability) was injected through the tympanic membrane using a Hamilton syringe. Rats were then placed on their side in their home cage and allowed to wake up. During recording sessions (one hour before and 1, 3, 5, 7, 24, 48 and 168 h after the lesion), the IMU was attached to the rat’s head and inertial signals were recorded for 45 min. To analyze head posture, low-pass filtered (<2 Hz) linear acceleration was used as an approximation of the gravity vector (see Fig. S5 and Supplementary Methods).

#### Fear extinction experiment

Four rats underwent a 7-day fear conditioning and extinction training procedure (see Supplementary Methods). Fear conditioning consisted of three white noise-footshock pairings (30 s white noise cue that co-terminated with a 1 s, 0.6 mA footshock) presented in the fear conditioning chamber (context A). During the next 5 days each rat was placed in the extinction chamber (context B) for a 34-minute extinction session during which 6 white noise cues were presented without footshock. Rats were equiped with the IMU and their behavior was recorded with a high definition video camera. The begining and end of each session, as well as each noise presentation, were synchronized with the emission of an IR signal recorded by the IMU via its IR synchronization channel (Fig. 6A), and were signaled in the video by the illumination of a red LED (see Supplementary Methods). This allowed us to realign IMU data with the videos.

An experimenter (BPG) well trained in observational behavioral inventory analyses used an instantaneous time-sampling procedure^38^,^44^ to characterize behavioral freezing from the video files. Freezing was defined as the absence of movement except for breathing. A regular beeping sound was synchronized with the video files so that observational judgements for “freezing” or “not freezing” were made every 2 s during the 30 s before, during and 30 s after each white noise presentation.

Automatic detection of immobility was performed by applying a simple threshold detection routine to the angular speed calculated from gyroscopic data (see Supplementary Methods). A “discrete” automatic scoring method was implemented to mimick the instantaneous time sampling procedure used by the observer. In this method, the average angular speed was calculated within a defined “observation” time window every 2 s, and a score (1 = immobility, 0 = movement) was attributed to each observation depending on whether the average angular speed was greater or smaller than a threshold value (Fig. S9A). For each noise presentation, this “discrete” automatic scoring method was applied to the same 90 s scored by the observer.

### Data acquisition and analysis

Inertial and videotracking data were acquired using custom programs written in LabVIEW. Data analysis was performed using routines written in R. Movies presented as Supplementary Material were generated using Python. To generate these movies, the same clock was used to timestamp inertial data (through the IR channel) and the acquisition of video frames under LabVIEW. Average values are given with their SD, unless stated otherwise.

## Additional Information

**How to cite this article**: Pasquet, M. O. *et al*. Wireless inertial measurement of head kinematics in freely-moving rats. *Sci. Rep.* **6**, 35689; doi: 10.1038/srep35689 (2016).

## Supplementary Material

Supplementary Information

Supplementary Movies S1

Supplementary Movies S2

Supplementary Movies S3

Supplementary Movies S4
