# Supplementary material for: Wireless inertial measurement of head kinematics in freely-moving rats
Source: Sci Rep. 2016 Oct 21;6:35689. doi: 10.1038/srep35689 (PMC5073323; doi:10.1038/srep35689)
Supplement: Supplementary Information [file srep35689-s1.pdf]

Supplementary information

Wireless inertial measurement of head kinematics in freely-moving rats

Matthieu O. Pasquet[1,a], Matthieu Tihy[2,a], Aurélie Gourgeon[2], Marco N. Pompili[3,4], Bill P. Godsil[3], Clément Léna[2] and Guillaume P. Dugué[2,b]

[1]Institut de Neurobiologie de la Méditerranée (INMED), Institut National de la Santé et de la Recherche Médicale (INSERM) U901, Marseille, France.
[2]Institut de Biologie de l'Ecole Normale Supérieure (IBENS), Centre National de la Recherche Scientifique (CNRS) UMR8197, Institut National de la Santé et de la Recherche Médicale (INSERM) U1024, Paris, France.
[3]Centre de Psychiatrie et Neurosciences (CPN), Institut National de la Santé et de la Recherche Médicale (INSERM) U894, Paris, France.
[4]Collège de France, Centre Interdisciplinaire de Recherche en Biologie (CIRB), Centre National de la Recherche Scientifique (CNRS) UMR7241, Institut National de la Santé et de la Recherche Médicale (INSERM) U1050, Paris, France.

[a]Equal contributions.

[b]Correspondence: `guillaume.dugue@ens.fr`

Contents

1 Supplementary Methods **2**
1.1 Data timestamping using IR synchronization . . . 2
1.2 Temporal performance measurements . . . 2
1.2.1 Transmission delays . . . 2
1.2.2 Measuring data sampling rate variations using outbound RF synchronization . . . 2
1.3 Surgical procedure for attaching magnets to the skull . . . 3
1.4 Analysis of head posture . . . 3
1.4.1 Orientation filter . . . 3
1.4.2 Changes in head posture following a unilateral vestibular lesion . . . 3
1.5 Fear conditioning experiments . . . 4
1.5.1 Apparatus/Method . . . 4
1.5.2 Fear conditioning and extinction procedures . . . 4
1.5.3 Manual scoring of behavioral freezing . . . 5
1.5.4 Analysis of gyroscopic data . . . 5
1.6 Comparison of the IMU with a motion capture system . . . 5
1.6.1 Sensitivity . . . 5
1.6.2 Resolution . . . 6

2 Supplementary Figures **6**

3 Supplementary Movies **18**

1 Supplementary Methods

1.1 Data timestamping using IR synchronization

IR synchronization was used to estimate precisely the absolute acquisition time of individual IMU data frames. To a first approximation, two distant IR synchronization events (for example at the begining and end of data acquisition) should provide reliable temporal limits within which all data frames could be linearly realigned. This method, however, assumes that the data sampling rate is regular. Because data acquisition is paced by the microcontroller's internal clock (instead of a crystal oscillator), we can expect that the data sampling rate will display some fluctuations over time. We therefore used a timestamping strategy based on the emission of regular IR signals (gated by the counter of a DAQ board; 1 Hz, duty cycle: 10%), allowing us to estimate variations of the local average sampling rate (the number of data frames acquired between two successive IR signals). This local sampling rate never displayed variations greater than one frame per second, indicating that the time between two acquisition cycles drifted by less than 3.3 ms over 1 s. Over several minutes, the average sampling rate displayed slow fluctuations of the order of ±0.5% (Fig. S3B). To calculate the acquisition time of individual data frames, we used the method depicted in Fig. S4. Data frames received from the IMU were realigned by assigning the time of the first and last counter ticks to the corresponding IMU data frames, and by linearly redistributing all other IMU data points within these limits. Because of the fluctuations in the IMU sampling rate, data points corresponding to the detection of IR events did not coincide with the occurrence of the corresponding counter ticks. This misalignment (lag) never exceeded ±200 ms (measured for each IR event in 7 recording sessions of 45 min). By fitting a spline function to the time course of the lag, a lag value could be calculated for each data frame. Linearly interpolated acquisition times could then be corrected by subtracting this value.

1.2 Temporal performance measurements

1.2.1 Transmission delays

To measure the delay between mechanical transduction by the sensor and data reception by the client software, the following procedure was used. The wireless IMU was attached to a metal plate connected to a 1.5 V battery through a 100 kΩ resistor (Fig. 2C). The battery was hit against the metal plate, creating a mechanical vibration measured by the IMU and a voltage difference across the resistor monitored at 20 kHz by the analog input of a DAQ board. The data transmitted by the IMU and the voltage across the resistor were recorded simultaneously using the same LabVIEW program. The shocks between the battery and the metal plate were visible as steps in the analog input channel and as brief oscillations in the linear acceleration data (Fig. 2C). For each step, the transmission delay was obtained by calculating the latency between the first points deviating significantly from baseline in both signals (red points in Fig. 2C).

For comparison, the same measurement was performed with a wired IMU (built by mounting the same digital inertial sensor onto a custom PCB). In this experiment, the wired IMU was connected to the PC via a USB I^2C interface (USB-8451, National Instruments).

In addition to enabling the calculation of accurate acquisition times (see 1.1), the use of IR synchronization also allowed us to measure transmission delays by calculating the lag between the emission of IR signals and the reception of the first IMU data frames containing information regarding these signals. These measurements confirmed that transmission delays lie in the 30–120 ms range (Fig. S3A).

1.2.2 Measuring data sampling rate variations using outbound RF synchronization

To obtain accurate measurements of the fluctuations of the microcontroller's acquisition rate, we programmed the microcontoller to trigger the emission of an RF signal every 300 acquisition cycles, and recorded the output of our RF receiver at 20 kHz using the analog input channel of a DAQ board. These measurements showed that variations of the acquisition rate never exceeded ±0.1% (Fig. S3C).

1.3 Surgical procedure for attaching magnets to the skull

Rats received an injection of the opioid analgesic buprenorphine (0.05 mg/kg s.c.) and were placed in a stereotaxic apparatus (model 942, David Kopf Instruments) under isoflurane anesthesia. The rat's scalp was shaved and wiped with povidone-iodine followed by 70% ethanol. Lidocaine (2%) was injected subcutaneously before incising the scalp. The skull was gently scraped with a scalpel blade and cleaned with a 3% hydrogen peroxide solution. Burr holes were drilled (two over the frontal and four over the parietal bone plates) in order to insert skull screws (#0-80 x 3/32" stainless steel screws, Plastics One). A layer of self-curing dental adhesive (Super-Bond C&B, Sun Medical) was then deposited over the skull. Two disk neodymium magnets (S-06-03-N, Supermagnete.com) glued to a small piece of glass/epoxy sheet were positioned over the screws and fixed to the rat's head using self-curing acrylic resin (Pi-Ku-Plast HP36, Bredent). Finally the skin ridges were sutured in front and at the rear of the implant and the rats were allowed to recover in their home cage.

1.4 Analysis of head posture

1.4.1 Orientation filter

In order to isolate the gravitational component of linear acceleration, we implemented an orientation filter algorithm developped by Sebastian Madgwick [1]. This algorithm integrates gyroscope signals (angular velocity) in order to keep track of the absolute 3D orientation. Although small, the noise of gyroscope measurements introduces an accumulating error in the estimated orientation. This error is corrected using accelerometer measurements, that would provide a measure of gravity (and thus an absolute reference of orientation) in the absence of translational acceleration. In normal operating conditions, transient translational acceleration signals add with gravity in accelerometer measurements. The task of the algorithm is to perform the optimal fusion of gyroscope and accelerometer measurements to provide a single estimate of orientation. One key parameter in the algorithm is the rate at which the estimated gravity converges toward the raw linear acceleration in order to compensate for the drift of gyroscope signal integration. Because the horizontal heading direction (azimuth) is not constrained by the filter, it tends to drift over time. This, however, does not compromise the estimation of orientation relative to gravity (attitude). A non-drifting estimate of absolute orientation could be obtained using magnetometer data (not used here), that provide a measurements of the earth's magnetic field.

To obtain a reliable estimate of orientation, it is important to subtract the offsets of gyroscopes and accelerometers. In 3 recordings, we recorded periods of total immobility (IMU on a table) before attaching it to the rat and measuring the animal's movements. This period of immobility was used to measure gyroscope offsets (typically ranging 0.1–1.3 °/s), and to calculate accelerometer offsets. The calculation of accelerometer offsets is performed as follows. Well calibrated accelerometers should give a net acceleration $\|a\|$ of 1 g during immobility in any orientations ($\|a\| = \sqrt{a_x^2 + a_y^2 + a_z^2}$, i.e. the magnitude of the acceleration vector a defined by a_x, a_y and a_z). Therefore accelerometer offsets can be calculated by minimizing $1 - \|a\|$ using data recorded from an immobile IMU in different orientations. Our calculations yielded offset values ranging 0.001–0.03 g.

For every time point in the recording, the output of the orientation filter is a quaternion representation of a rotation matrix R_θ that brings the object from an arbitrary initial orientation to the current estimated orientation. By choosing an upright initial orientation (z sensor axis aligned with gravity), it is possible to track the orientation of gravity in the sensor's coordinates (G) by applying the converse rotation: $G = R_{-\theta} \cdot z_i$, where $R_{-\theta}$ is the matrix corresponding to the converse rotation and $z_i = (0, 0, 1)$ is the initial orientation of the z axis. Fig. S5 shows that G follows the slow fluctuations of the measured linear acceleration (Fig. S5A) and that G accounts for most of the linear acceleration below 1–2 Hz (Fig. S5B). This shows that low-pass filtered linear acceleration can be used as an approximation of gravity.

1.4.2 Changes in head posture following a unilateral vestibular lesion

The low-pass filtered (< 2 Hz) linear acceleration vector (a_{lp}) was used as an approximation of gravity (see 1.4.1). To obtain a density map of the different orientations of a_{lp} encountered during a recording, the cartesian coordinates of a_{lp} were transformed into polar coordinates (longitude and latitude), and a 2D histogram of these polar coordinates was calculated. The bins of this 2D histogram represent quadrangles with different areas when represented on a sphere. In order to plot this histogram on a sphere, the number of occurrences of a_{lp} coordinates falling within a specific bin was normalized by the corresponding quadrangle area (Fig. 5B,C). Normalized histogram data were then color coded using a logarithmic scale and wrapped onto a

sphere using functions of the `sphereplot` R package. To obtain meaningful a_{lp} orientation density maps (i.e. representing the range of head postures), we took care to remove situations in which animals would lean their head against the walls of the arena or lie down on the floor for an extended period of time. Such situations could potentially artificially shift a_{lp} orientation density maps and bias our calculation of an average head posture. To achieve this, we excluded from the analysis the periods during which the IMU LEDs were less than 2 cm away from the walls, and/or the instantaneous head angular speed was less than 20 °/s.

The following procedure was used to obtain 2D density maps (Fig. S5 and S6). 2000 points were uniformly distributed on a sphere or radius 1. For a given recording, our algorithm counted the number of times $a_l p$ was aligned with each of these positions (±2°), yielding a vector N with 2000 values. A Delaunay triangulation was then performed around these points to obtain a set of 2000 polygons distributed over the sphere. A 2D projection of these polygons was obtained using a transformation that preserved their respective areas (using the `mapproj` R package). A color code was used to represent the value of N (on a logarithmic scale) for each location. Correlations between density maps from different rats were calculated by computing the Pearson correlation coefficient between the corresponding vectors N.

For the unilateral vestibular lesion experiment, head posture was reconstituted in 3D using the mean low-pass filtered linear acceleration vector ($\overline{a_{lp}}$) calculated for each recording session (Fig. 5c). A 3D model of a rat's skull [2] was rotated to align $\overline{a_{lp}}$ with the vertical (z) axis of a right-handed reference frame, whose x axis was positioned such that it falls within the plane defined by z and the animal's naso-occipital axis. The roll tilt angle was defined as the angle between the head-vertical axis and this plane. The pitch angle was defined as the angle between the naso-occipital axis and the horizontal plane.

1.5 Fear conditioning experiments

1.5.1 Apparatus/Method

Rats underwent fear conditioning and extinction training in each of two distinct chamber configurations. The fear conditioning chamber (Context A) consisted of a hexagonal-shaped transparent Plexiglas box with the dimensions of $37 \times 45 \times 37$ cm (L×H×W) with a grid floor composed of 25 stainless steel rods (0.6 cm in diameter) that were connected to a constant-current scrambled shock generator (ENV-414, Med Associates, USA). This chamber was scented with a 1% ammonium hydroxide solution in the collection pan below the grid floor. The extinction chamber (Context B) was a rectangular-shaped box with the dimensions of $40 \times 45 \times 40$ cm (LxHxW), which had opaque grey Plexiglas rear and side walls, a transparent Plexiglas front wall, and a black plastic insert covering the floor. In addition, to ensure that the rats were fully visible to the camera, a black plastic ramp insert (40×18 cm) was installed, which slanted up from the middle of the floor to the front of the chamber. This chamber was scented with a 1% acetic acid solution. During the behavioral sessions the sound and shock generators were used to deliver white noise cues (30 s; 70 dBA) and/or footshocks (1 s; 0.6 mA) to the animals via a computer-controlled interface (Med Associates, USA). Each context was maintained inside a larger, insulated plastic cabinet that provided a degree of sound attenuation from ambient external noise. A speaker attached to a sound generator (ENV-230, Med Associates, USA) was mounted above the context (48 cm from the floor). To ensure high quality video acquisition, a 150 W halogen spotlight was used to illuminate each context from above (754 lux). The spotlight's fan and the noise emanating from the computer-controlled interface provided background noise (48 dBA).

Rat behavior was recorded with a high-definition video camera (Sony Handycam HDR-CX280) positioned 30 cm from the front wall of the testing chamber. It captured images that were converted into mp4 video files (1920×1080 pixels images captured at 50 frames per second) that were used during subsequent observational behavioral analyses. The video resolution allowed the observer to visualize motion in the rat's vibrissae easily. A red LED that was visible to the camera, but not to the rat, was positioned outside of each testing chamber, and its illumination pattern was used to synchronize the timing of the video data with the sensor measurements.

1.5.2 Fear conditioning and extinction procedures

Rats were handled daily for 7 days prior to the magnet implantation procedure. After 14 days of recovery they were handled again for another 7 days prior to the start of the 7-day fear conditioning and extinction procedures. On day 1 each rat underwent a 34-minute habituation to Context A. On day 2, each rat returned to context A where it was fear conditioned with three white

noise-footshock pairings (30 s white noise cue that co-terminated with the 1 s 0.6 mA footshock). During this session, the onset of the white noise cues occurred at 300, 630, and 960 s after the initiation of the control program. On days 3–7 each rat was placed in context B for a 34-minute extinction session during which 6 white noise cues were presented without footshock at 300, 630, 960, 1290, 1620, and 1950 s after initiation of the control program. On day 1 and days 3–7 the sensor device was attached to the animal's head immediately before the behavioral session. The sensor was not installed on the fear conditioning day, however, to ensure device integrity.

1.5.3 Manual scoring of behavioral freezing

An experimenter (BPG) well trained in observational behavioral inventory analyses used an instantaneous time-sampling procedure [3] to characterize behavioral freezing from the video files. Freezing was defined as the absence of movement except for that required for breathing. A regular beeping sound was synchronized with the video files so that observational judgements for "freezing" or "not freezing" were made every 2 seconds during the 30 s before, during and 30 s after each white noise presentation.

1.5.4 Analysis of gyroscopic data

In these recordings, the sensitivity ranges of the IMU were set to their minimum (±2 g and ±250 °/s) in order to achieve the highest resolution for small movements. Data obtained from the 3 gyroscopes (ω_x, ω_y and ω_z) provide the coordinates of the instantaneous 3D angular velocity vector ω, whose magnitude $\|\omega\|$ is the instantaneous angular speed. The angular speed signal was first smoothed by applying a low-pass filter (5 Hz), in order to remove rapid transient threshold crossings. The state of the animal (immobile or not) was then assessed by running a simple threshold crossing detection routine on the low-pass filtered angular speed. In a first type of analysis, ("discrete" automatic scoring), the average angular speed ($\|\overline{\omega}\|$) was assessed within a defined time window every 2 s, and a score (immobility or not) was attributed to each observation window depending on wether $\|\overline{\omega}\|$ was greated or smaller than the threshold (Fig. S6A). This method aimed at mimicking the instantaneous time sampling procedure implemented by the observer. In a second type of analysis, the threshold was simply applied to the whole angular speed trace and the fraction of immobility (% of the points below threshold) was calculated (Fig. S6B).

1.6 Comparison of the IMU with a motion capture system

In this section, we detail a number of calculations that were used to compare our IMU with a video motion capture system for the detection of head rotations.

1.6.1 Sensitivity

We simulated a situation in which a high rate, high resolution camera records the movements of a rat over a 1 m^2 square area. Two reflectors (R1 and R2) are placed on the head of a rat, separated by a distance d (Fig. S11A). For d to be greater than the head's length ($\simeq$ 3 cm), the two reflectors can be placed at the two extremities of a thin bar fixed to the skull. Here we postulate an ideal situation in which the video system detects the exact position of the centers of R1 and R2. The simulated movement is a simple yaw rotation centered around R1, that brings R2 to a new position located at a distance p toward the left (Fig. S11B). The smallest detectable rotation produces a lateral displacement of R2 corresponding to one pixel ($p = 0.25$ mm for a 4000 $\times$ 4000 pixel camera and $p = 1$ mm for a 1000×1000 pixels camera, assuming $d = 5$ cm). This corresponds to a minimum rotation angle θ_{min} of $\arctan(\frac{p}{d})$ (i.e. $\theta_{min} = 0.29°$ for a 4000×4000 pixel camera and $\theta_{min} = 1.15°$ for a 1000×1000 pixel camera).

On the IMU side, the smallest detectable rotation can be calculated based on the noise of gyroscopic measurements. This noise was quantified by recording inertial data from an immobile IMU placed on a mechanically-isolated platform, and configured to use the highest gain possible (±250 °/s). The SD of the measured signal ranged 0.10–0.12 °/s (Fig. S11C), meaning that a rotation speed ω greater than 0.4 °/s ($\simeq$ 3.5 SD) should be detectable. Note that this angular speed is extremely slow (one full turn in 15 min).

The graph in Fig. S11D represents a 2D space where the y axis is the rotation angle θ, and the x axis is the duration of the rotation. Given the above calculations, all rotations with $\theta > 0.29°$ (for a 4000×4000 pixel camera) or with $\theta > 1.15°$ (for a

1000 × 1000 pixel camera) are detectable by the video system (solid horizontal lines in Fig. S11D). As calculated above, all rotations with an angular speed $\omega > 0.4\,°/\mathrm{s}$ can be detected by the IMU (lower oblique dashed line in Fig. S11D). The gray area highlights rotations that can be considered as physiologically-relevant for studying rat behavior (0.2–20 Hz). The graph shows that the IMU can detect rotations that the video system can not discriminate (green area in Fig. S11D: $\theta < 0.29°$ or $1.15°$ depending on the resolution of the camera, and $\omega > 0.4\,°/\mathrm{s}$). Conversely, the video system can detect slow rotations that the IMU can not capture (red area in Fig. S11D: $\theta > 0.29°$ or $1.15°$ and $\omega < 0.4\,°/\mathrm{s}$). It is important to note that the regions where one system performs better than the other correspond to rotations that lie outside or at the edge of the physiologically-relevant range. Therefore we conclude that both systems can achieve a level of sensitivity (the ability to detect small head rotations) that is adapted to the study of behaving rats.

1.6.2 Resolution

In the following simulation, the rotation was a 5° yaw rotation executed in 0.1 s, corresponding to the type of gyroscopic signal acquired in freely behaving rats (see Movie S2). Fig. S11E represents the absolute yaw angle and Fig. S11F shows the expected angular measurement achieved with a video tracking system equiped with a 4000 × 4000 pixel camera functioning at 100 Hz. Fig. S11G shows the expected gyroscopic signal ω acquired at 300 Hz, calculated using the noise level measured above. Fig. S11H shows an estimation of ω calculated from the angles measured by the video system. These graphs show that for a typical small head rotation, the IMU provides a much better resolution of angular velocity than a motion tracking system.

2 Supplementary Figures

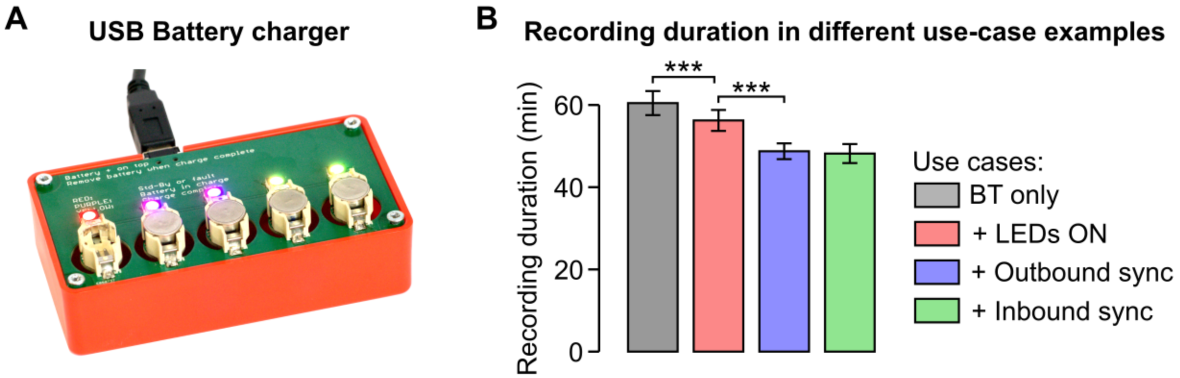

Figure S1: Battery charger and battery life. ***A***, Photograph of the USB battery charger. The charger contains 5 independent charging positions. Each position features a tell-tale whose color indicates the charging status (red: standby or fault; purple: battery in charge; yellow: charge complete). ***B***, Average battery life for different use cases. The maximal duration of recordings, using fully charged batteries, was measured in different configurations, as indicated in the legend (n = 17 measurements for each condition, performed with 17 different batteries). Our measurements yielded the following average recording durations: BT only (Bluetooth transmission only): 3627 ±173 s; + LEDs (LEDs constantly on): 3374 ±150 s; + Outbound sync. (RF outbound signal delivered at 300 Hz, duty cycle = 10%): 2924 ±112 s; + Inbound sync. (constant IR inbound signal): 2891 ±136 s. Asterisks indicate statistically significant differences (***: $p < 0.001$, unpaired t-test). Error bars represent the SD. Overall, the strongest determinant of battery life is the usage of the RF emitter (reduction of 13% when RF power is emitted 10% of the time).

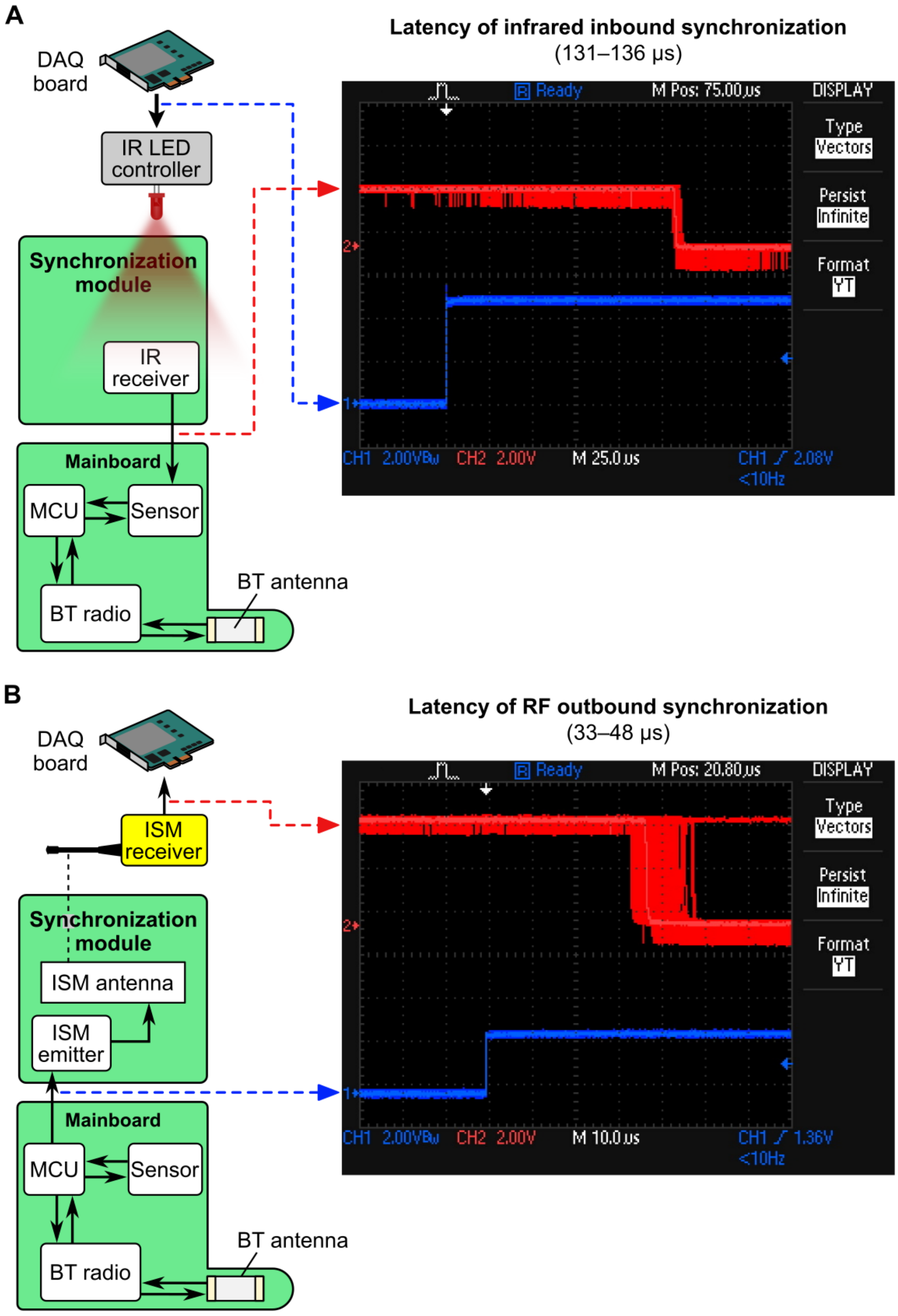
A
DAQ board
IR LED controller
Synchronization module
IR receiver
Mainboard
MCU
Sensor
BT radio
BT antenna
Latency of infrared inbound synchronization
(131–136 μs)
Ready
M Pos: 75.00μs
DISPLAY
Type
Vectors
Persist
Infinite
Format
YT
CH1 2.00VBw CH2 2.00V M 25.0μs CH1 2.08V
<10Hz
B
DAQ board
ISM receiver
Synchronization module
ISM antenna
ISM emitter
Mainboard
MCU
Sensor
BT radio
BT antenna
Latency of RF outbound synchronization
(33–48 μs)
Ready
M Pos: 20.80μs
DISPLAY
Type
Vectors
Persist
Infinite
Format
YT
CH1 2.00VBw CH2 2.00V M 10.0μs CH1 1.36V
<10Hz

Figure S2: Latencies of inbound and outbound synchronization. ***A***, For measuring the latency of inbound synchronization, square pulses (1 Hz, duty cycle: 10%) were generated using a DAQ board and fed to the gate input of the IR LED module. The oscilloscope screenshot (right) shows 900 pulses aligned on their rising front (blue traces) and the voltage measured simultaneously at the output of the IR receiver on the IMU (red traces). The IR receiver output switches from 2.7 to 0 V when it detects an appropriate optical stimulus and this signal is inverted before reaching the sensor's synchronization input (not shown on circuit diagram on the left). The observed latency ranged from 131 to 136 µs. ***B***, For measuring the latency of outbound synchronization, the microcontroller was configured to deliver RF pulses every 300 acquisition cycles (duration of pulses high state: 150 cycles). The oscilloscope screenshot (right) shows 900 voltage traces measured at the input of the ISM emitter on the IMU (blue traces) and the voltage measured simultaneously at the output of the RF receiver (red traces). The observed latency ranged from 33 to 48 µs. The receiver was positioned 60 cm away from the IMU.

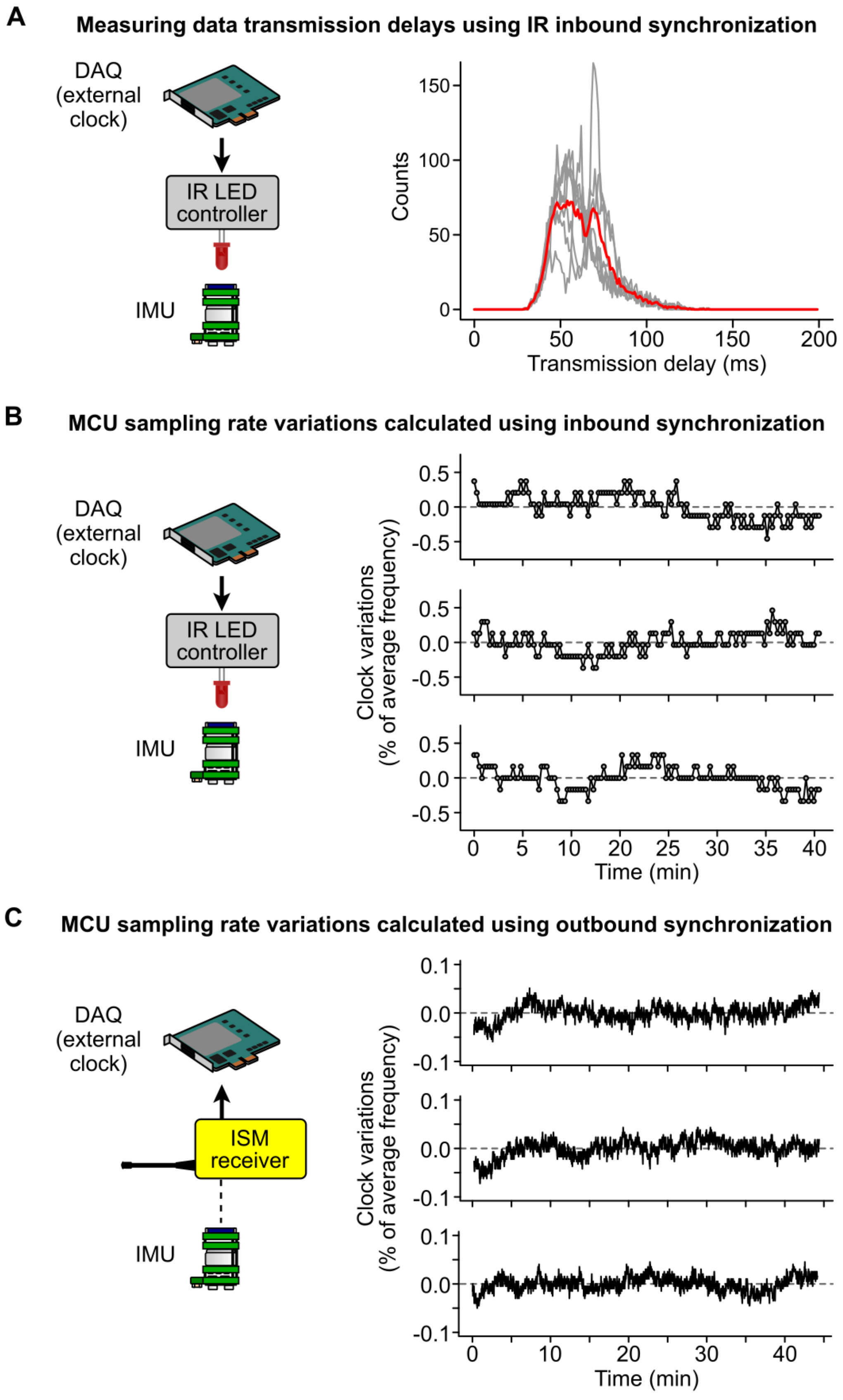
A
Measuring data transmission delays using IR inbound synchronization
DAQ
(external
clock)
IR LED
controller
IMU
Counts
Transmission delay (ms)
B
MCU sampling rate variations calculated using inbound synchronization
Clock variations
(% of average frequency)
Time (min)
C
MCU sampling rate variations calculated using outbound synchronization
ISM
receiver

Figure S3: Estimating the delay of Bluetooth data transmission and the regularity of data acquisition using synchronization functions. ***A***, Histograms of Bluetooth transmission delays calculated from 7 recording sessions (gray traces), and their average (red). The transmission delay was measured by connecting the counter of a DAQ to the IR LED controller, and by calculating the lag between individual counter ticks and the time of reception of the corresponding inbound synchronization events in the IMU data stream. Each session contained 2700 pulses delivered by the counter at 1 Hz (duty cycle: 10%). All histograms have a bin of 1 ms. The transmission delays measured using this method are comparable to those obtained in Fig. 2d. ***B***, Fluctuations of the IMU data acquisition rate estimated using IR synchronization. The graph contains 3 example traces from 3 different recording sessions. In this experiment, regular IR signals were emitted at 1 Hz and the IMU acquisition frequency was calculated based on the number of frames acquired between two successive IR synchronization events. ***C***, Fluctuations of the IMU data acquisition rate estimated using outbound synchronization. The graph contains 3 example traces from 3 different recording sessions. In this experiment, RF power was emitted by the IMU every 300 frames and the ouput of the RF receiver was monitored at 20 kHz. The calculated data acquisition rate displayed slow fluctuations comparable to those observed in *B*, and small rapid variations which correspond to the jitter of the RF synchronization channel (see Fig. S2B).

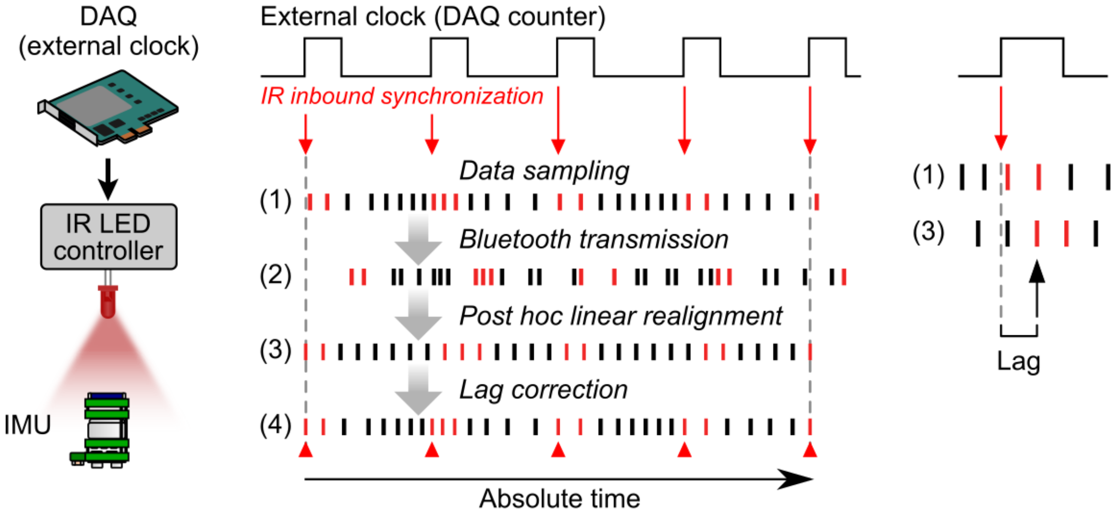

Figure S4: Strategy for calculating the acquisition times of IMU data frames using IR synchronization. The counter of a DAQ board was used to gate the emission of IR signals that were detected by the IMU (left). In the middle panel, individual IMU data frames are represented by vertical bars. Red bars represent data frames acquired in the presence of an IR signal. The following strategy was used to calculate the acquisition times of IMU data frames. Inertial data acquired by the IMU (step 1: *Data sampling*) and transmitted via Bluetooth with a variable delay (step 2: *Bluetooth transmission*) were realigned by assigning the time of the first and last counter ticks to the corresponding IMU data frames, and by evenly redistributing all data frames within these limits (step 3: *Post hoc linear realignment*). The misalignment (lag) between counter ticks and the corresponding IMU data frames was then calculated (inset on the right). Lag correction was performed by fitting a spline function to the time course of the lag and then subtracting spline values from the times of IMU data points (step 4: *Lag correction*). In practice, the period of the external clock has to be significantly shorter than the time scale of the fluctuations in the IMU's sampling rate. In the illustration, fluctuations of the sampling rate and transmission delay are exaggerated for the sake of the demonstration.

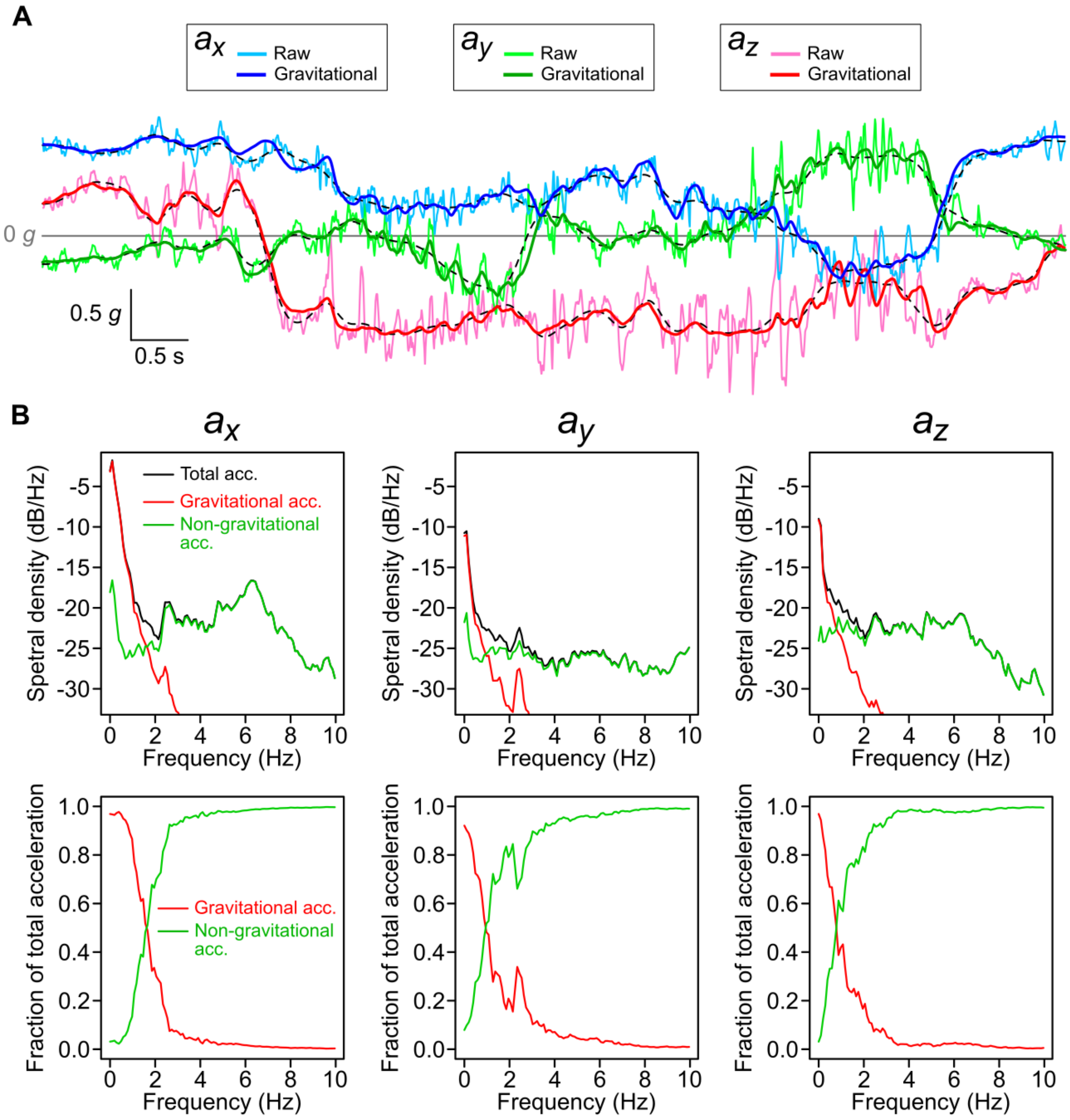

Figure S5: Estimation of the gravitational component of linear acceleration using an orientation filter algorithm. An orientation filter algorithm was used in 3 recordings to estimate the gravitational component of linear acceleration (see 1.4.1 for details). ***A***, Example trace recorded from a freely moving rat, showing the raw linear acceleration (light colors) and the predicted gravitational acceleration (strong colors). Dashed lines represent the low-pass filtered acceleration (< 2 Hz). ***B***, Top: average spectral density (in dB) of the total (black), gravitational (red) and non-gravitational (green) components of linear acceleration, calculated from 3 recordings. Bottom: average fraction of total spectral density carried by the gravitational (red) and non-gravitational (green) components of linear acceleration, calculated from 3 recordings.

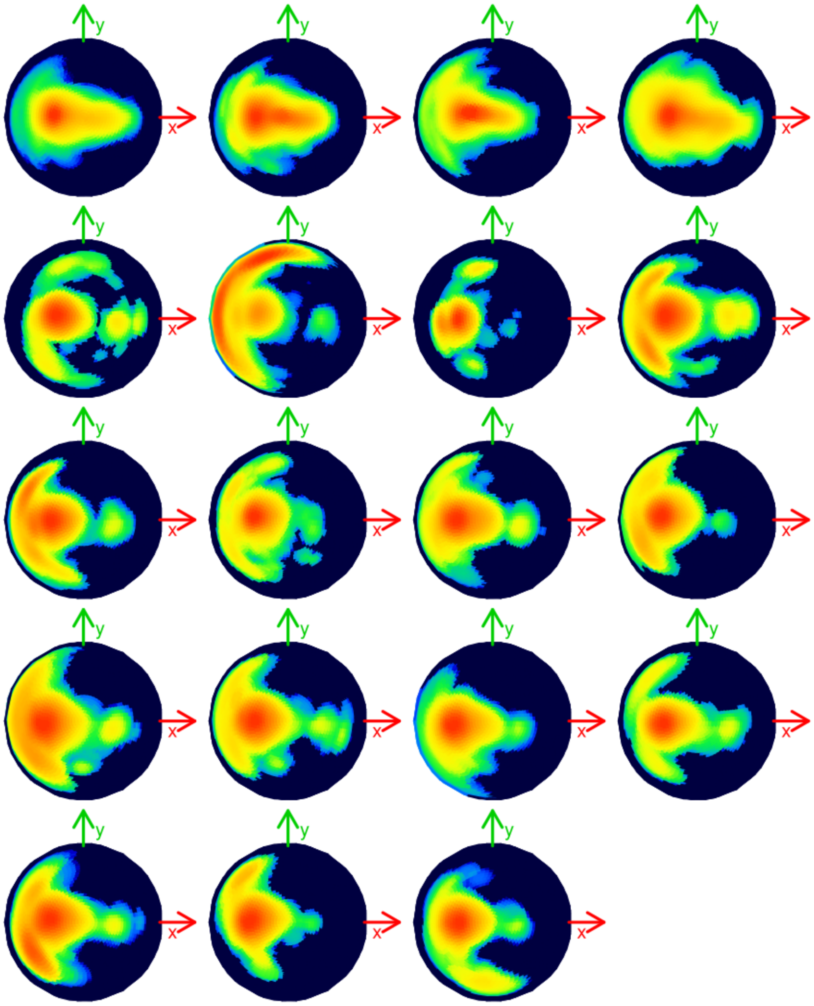

Figure S6: Examples of "gravity orientation density maps" calculated for 19 different rats. The plots are 2D projections of spherical orientation maps (such as the one displayed in Fig. 5b), where the North pole of the sphere (z axis in the sensor's reference frame) is at the center (see 1.4.2 for a description of the calculation of these maps). The color code represents the frequency at which the gravity vector was oriented in a specific direction (logarithmic scale) in the sensor's coordinates. The red and green arrows show the directions of the x and y axes in the sensor's reference frame. Recordings lasted between 30 and 45 min.

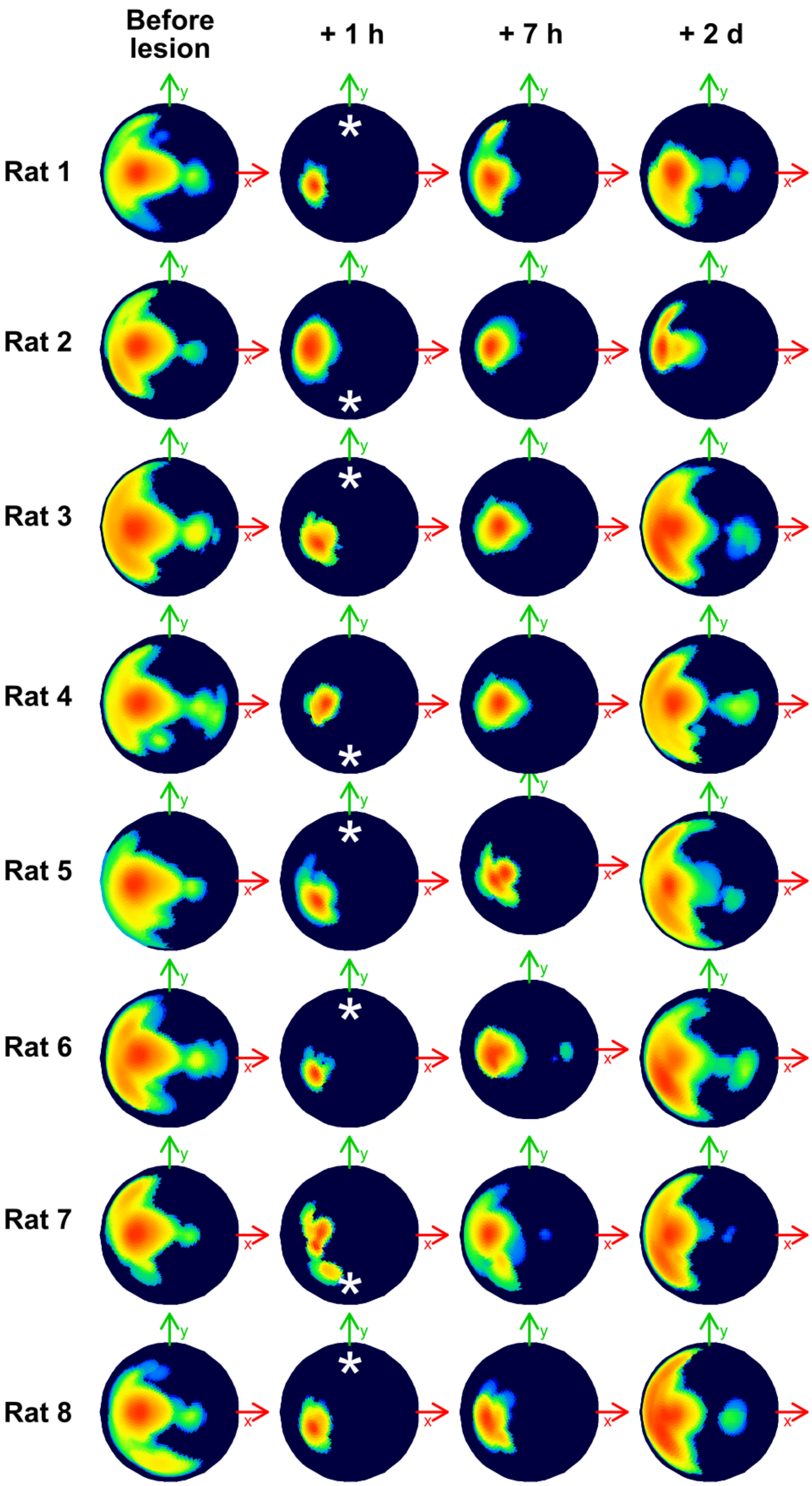

Before lesion
+ 1 h
+ 7 h
+ 2 d
Rat 1
Rat 2
Rat 3
Rat 4
Rat 5
Rat 6
Rat 7
Rat 8

Figure S7: Gravity orientation density maps before and after unilateral vestibular lesion. The maps for all 8 rats that underwent a unilateral vestibular lesion are shown, before and at various intervals after the lesion (see 1.4.2 for a description of the calculation of these maps). White asterisks indicate the lesion side.

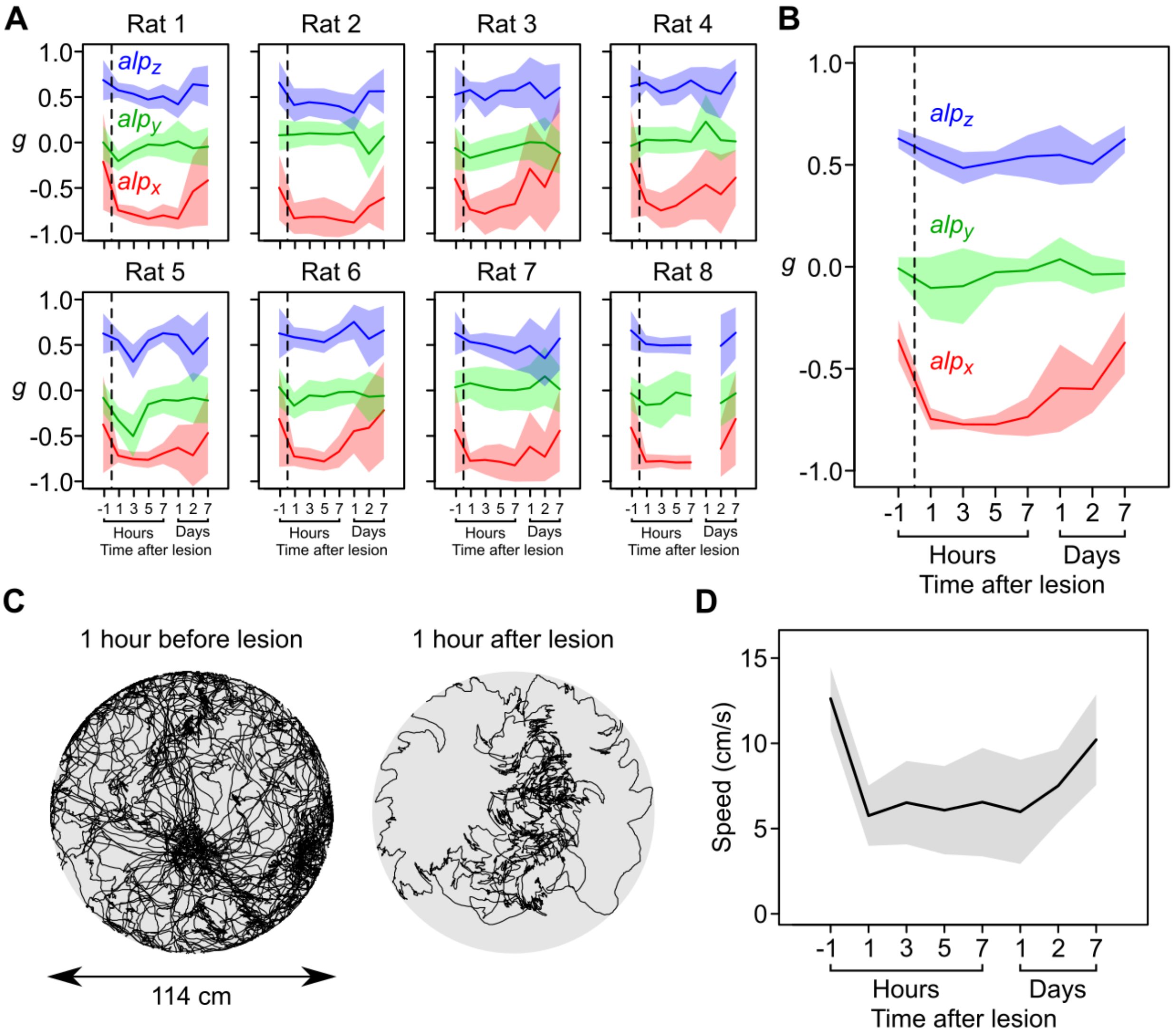

Figure S8: Changes in head posture and overall mobility after a unilateral vestibular lesion. ***A***, Average values (±SD as shaded area) of the low-pass filtered (< 2 Hz) linear acceleration signals (alp_x, alp_y and alp_z) for all rats, one hour before and at various time intervals after the lesion. ***B***, Average values (±SD as shaded area) of alp_x, alp_y and alp_z across rats. ***C***, Movement trajectories within the circular arena obtained for one rat for the sessions preceding (1 h before) and following (1 h after) the lesion. Trajectories were computed online by tracking the 3 LEDs present on top of the IMU. ***D***, Average speed (±SD as shaded area) across rats, calculated using LED videotracking data, and plotted for all recording sessions.

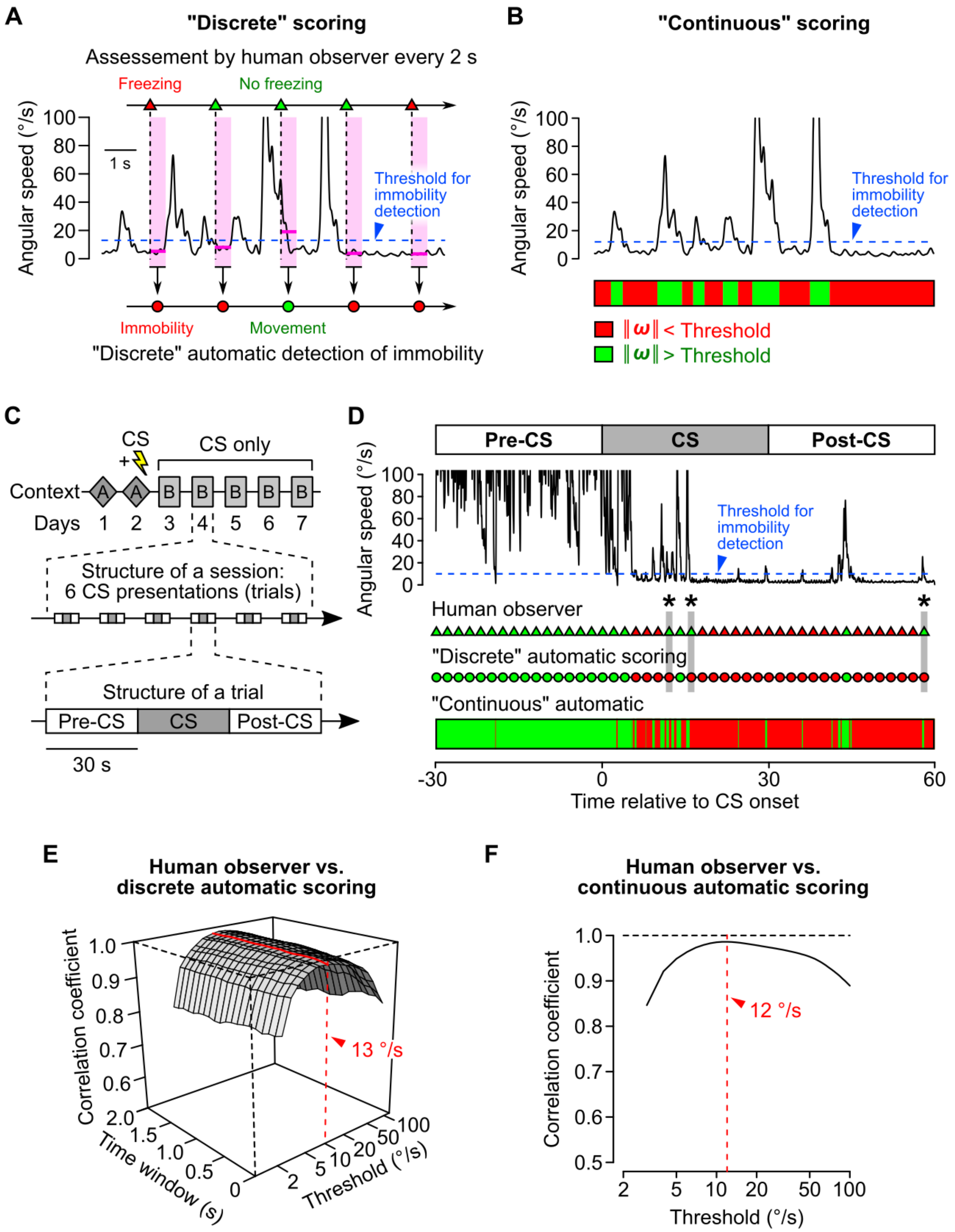
A
"Discrete" scoring
Assessement by human observer every 2 s
Freezing
No freezing
Angular speed (°/s)
1 s
Threshold for immobility detection
Immobility
Movement
"Discrete" automatic detection of immobility
B
"Continuous" scoring
Angular speed (°/s)
Threshold for immobility detection
‖ω‖ < Threshold
‖ω‖ > Threshold
C
CS +
CS only
Context
Days 1 2 3 4 5 6 7
Structure of a session: 6 CS presentations (trials)
Structure of a trial
Pre-CS
CS
Post-CS
30 s
D
Pre-CS
CS
Post-CS
Angular speed (°/s)
Threshold for immobility detection
Human observer
"Discrete" automatic scoring
"Continuous" automatic
Time relative to CS onset
E
Human observer vs. discrete automatic scoring
Correlation coefficient
13 °/s
Time window (s)
Threshold (°/s)
F
Human observer vs. continuous automatic scoring
Correlation coefficient
12 °/s
Threshold (°/s)

Figure S9: Methods for automatically scoring behavioral freezing in a fear conditioning experiment. ***A***, Drawing depicting the observational and automatic instantaneous time sampling procedures. Freezing was assessed by a trained observer (filled triangles) every 2 s using a regular beeping sound. "Discrete" automatic scoring was performed by calculating the average angular speed $\|\overline{\varpi}\|$ (horizontal purple lines) within defined time windows (purple bars) aligned to the observer's judgement times (vertical dashed lines), and by comparing its value with a threshold (immobility if $\|\overline{\varpi}\| <$ threshold, represented as a dashed blue line). ***B***, Drawing depicting the "continuous" automatic scoring technique, in which all data points are compared with a threshold. ***C***, Schematic of the experiment. Daily extinction sessions contained 6 CS presentations. Each CS presentation was scored by considering a *trial*, defined as a period encompassing the CS presentation as well as the 30 s before (Pre-CS) and after (Post-CS). ***D***, Example of a trial showing the angular speed trace and the scores obtained by the human observer and the two automatic scoring techniques. Note the occasional discrepancies (asterisks) between the scores obtained by the observer and the "discrete" automatic method. ***E***, Correlation coefficients between the *per trial* immobility scores obtained by the observer and the "discrete" automatic scoring algorithm, plotted as a function of the algorithm's parameters (width of observation time window and threshold value in log scale). For most time window values, the correlation was maximal for a threshold of 13 °/s (red line). ***F***, Correlation coefficients between the observer's *per trial* immobility scores and the average fraction of time spent below threshold calculated for each trial by the "continuous" automatic scoring algorithm, plotted against the algorithm's threshold value (log scale).

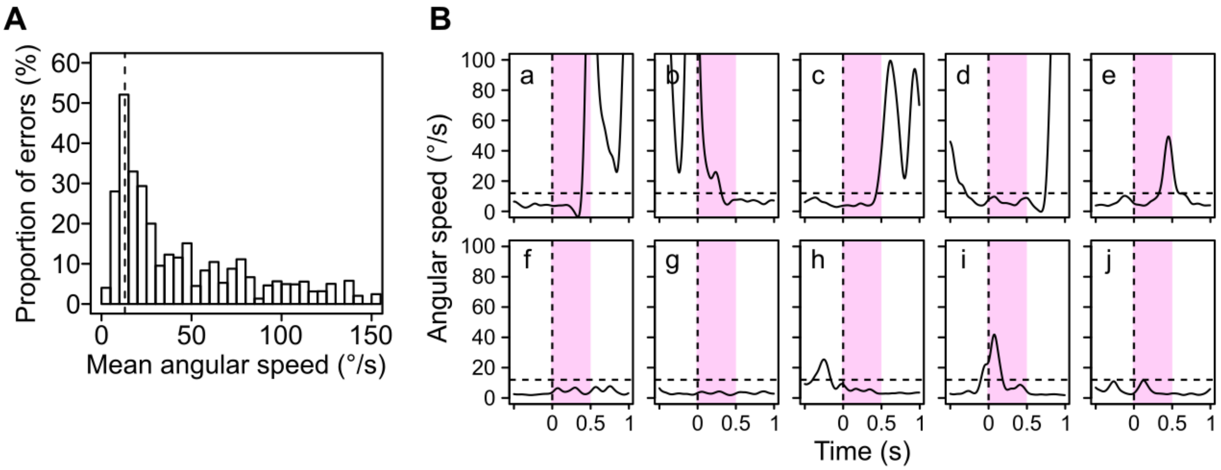

Figure S10: Analysis of the discrepancies between the scores obtained by the observer and the "discrete" automatic scoring method. ***A***, Fraction of scores with a mismatch plotted as a function of the local average angular speed calculated by the algorithm (using a time window of 0.5 s). The highest fraction of mismatch (up to 50% of observations) was found for near-threshold angular speeds. The threshold (13 °/s) is represented by a vertical dashed line. ***B***, Randomly selected angular speed traces corresponding to situations with a scoring discrepancy. The vertical dashed line represents the moment that beep (used by the observer) is emitted at the start of a 2-second bin. The purple rectangle marks the time interval employed for the "discrete" automatic scoring method (threshold = 13 °/s, horizontal dahed lines). In most cases, the time window fell at the begining or end of a head movement (a–e and h,i).

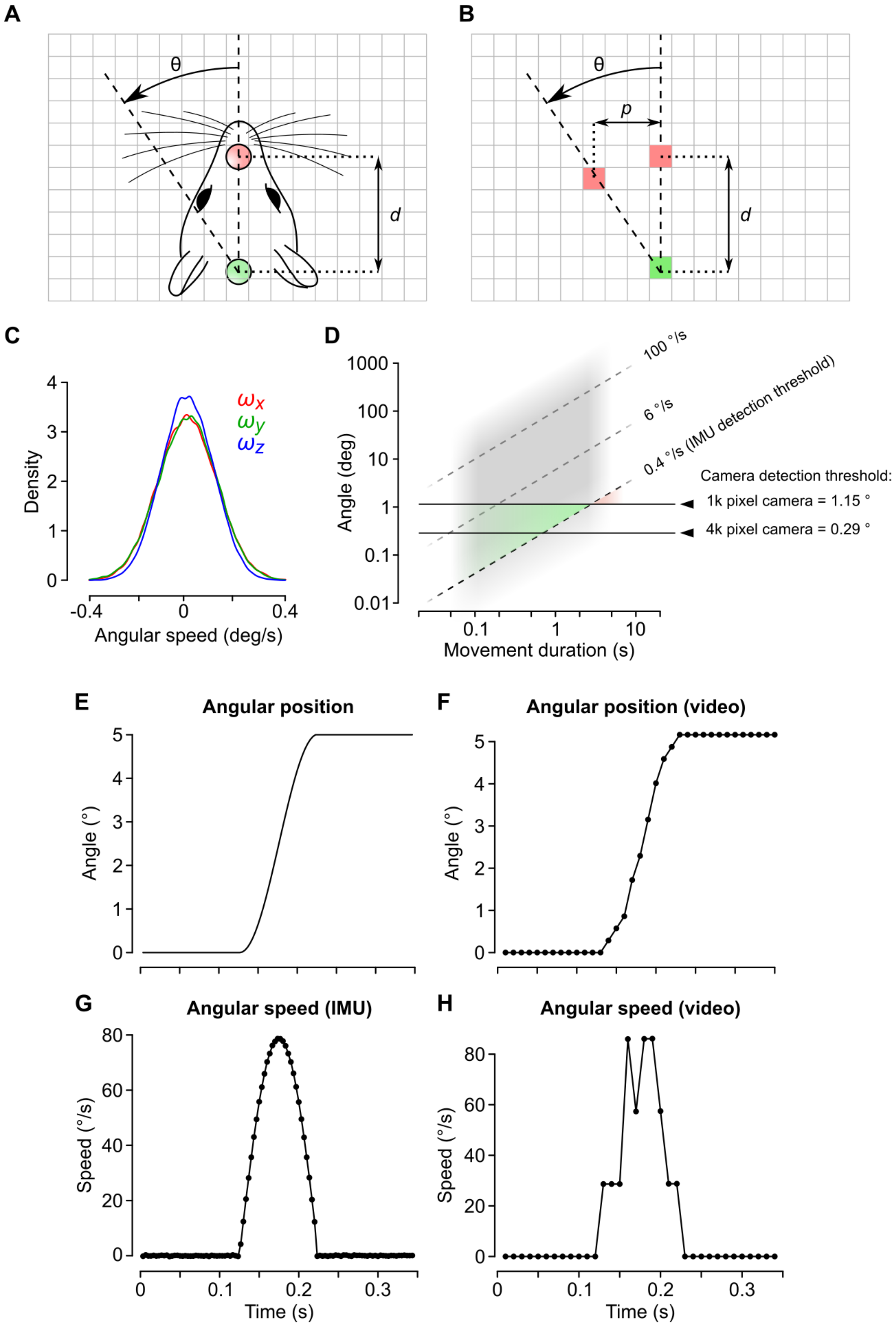
A
B
θ
p
d
C
Density
Angular speed (deg/s)
ω_x
ω_y
ω_z
D
100 °/s
6 °/s
0.4 °/s (IMU detection threshold)
Camera detection threshold:
1k pixel camera = 1.15 °
4k pixel camera = 0.29 °
Angle (deg)
Movement duration (s)
E
Angular position
Angle (°)
F
Angular position (video)
G
Angular speed (IMU)
Speed (°/s)
Time (s)
H
Angular speed (video)

Figure S11: Comparison of the IMU with a motion capture system for the detection of head rotations. To compare both systems, we simulated a simple yaw rotation and assumed that the video system is used to record the movements of a rat on a 1 m^2 square area, using two reflectors placed on its head. We assumed that the system is equiped with a high resolution camera (1000 × 1000 pixel or 4000 × 4000 pixel) functioning at 100 Hz. ***A-B***, Drawing depicting the simulated head rotation (see 1.6.1 for detailed explanations). ***C***, Centered density histogram of the angular velocities measured using the IMU placed on a mechanically isolated platform. ***D***, Graph summarizing the types of rotations that will be preferentially detected by our IMU or by a video motion tracking system (see 1.6.1 for detailed explanations). ***E-H***, Simulation comparing the resolution of both systems for the measurement of a small head rotation (see 1.6.1 for detailed explanations).

3 Supplementary Movies

Movie S1. Inertial signals recorded during active exploration. The upper left panel is a flow chart displaying the linear acceleration (a_x, a_y and a_z) and angular velocity signals (ω_x, ω_y and ω_z). The low-pass filtered (< 2 Hz) linear acceleration (a_{lp}, strong colors) is superimposed on the raw linear acceleration (light colors). The bottom left panel shows the current orientation of a_{lp} (green circle with a black border) on a spherical "orientation density map" (see Fig. 5B and 1.4.2). In order to visualize all possible orientations of the vector, the density map is represented using a Mollweide projection. The trajectory of a_{lp} orientations over the last second is represented by a thick green line. The right panel shows a movie of the rat acquired with a hand-held camera. The same clock was used to timestamp inertial data (through the IR channel) and the acquisition of video frames. Therefore the flow chart displaying inertial data (top left) and the current a_{lp} orientation (bottom left) are synchronized with the video of the rat. In the flow chart (top left), the dashed line corresponds to the time of the current video frame. The movie shows large increases in head angular velocity (up to ±500 °/s, typically lasting 200 ms, as well as episodes of rhythmic activity at around 10 Hz.

Movie S2. Rhythmic activity during active exploration. See the legend of Movie S1 for a description of the panels. This movie shows an episode of active exploration in slow motion (slowed 3-fold). This episode contains pronounced 10 Hz oscillatory activity, mainly visible in gyroscopic data, that is apparently not correlated with gait and might reflect active sniffing.

Movie S3. Inertial signals during grooming. See the legend of Movie S1 for a description of the panels. This movie shows an episode of head and body grooming. Head grooming is associated with pseudo-rhythmic activity, while body grooming is associated with large oscillations of both accelerometer and gyroscope signals at around 4 Hz. During body grooming, the low-pass filtered linear acceleration vector points towards characteristic regions of its orientation density map.

Movie S4. Inertial signals during rearing. See the legend of Movie S1 for a description of the panels. This movie shows episodes of rearing in slow motion (slowed 3-fold), during which the low-pass filtered linear acceleration vector follows a characteristic trajectory along the midline of its orientation density map.

References

[1] S. O. H. Madgwick, A. J. L. Harrison, and R. Vaidyanathan. "Estimation of IMU and MARG orientation using a gradient descent algorithm". *IEEE International Conf. on Rehabilitation Robotics*. IEEE, 2011, pp. 1–7.

[2] B. M. Pohl et al. "3D printers may reduce animal numbers to train neuroengineering procedures". *International IEEE/EMBS Conference on Neural Engineering, NER* (2013), pp. 887–890.

[3] M. S. Fanselow. "Conditioned and unconditional components of post-shock freezing." *The Pavlovian journal of biological science* 15.4 (1980), pp. 177–82.
